# Excitatory VTA to DH projections provide a valence signal to memory circuits

**DOI:** 10.1038/s41467-020-15035-z

**Published:** 2020-03-19

**Authors:** Yuan Han, Yi Zhang, Haram Kim, Viktoriya S. Grayson, Vladimir Jovasevic, Wenjie Ren, Maria V. Centeno, Anita L. Guedea, Mariah A. A. Meyer, Yixin Wu, Philipp Gutruf, Dalton J. Surmeier, Can Gao, Marco Martina, Apkar V. Apkarian, John A. Rogers, Jelena Radulovic

**Affiliations:** 1 Department of Psychiatry and Behavioral Sciences, Northwestern University, Chicago, IL 60611 USA; 2 Jiangsu Province Key Laboratory of Anesthesiology, Xuzhou Medical University, Xuzhou, 221004 Jiangsu China; 3 Department of Materials Science and Engineering, Northwestern University, 60208 Evanston, IL USA; 4 College of Engineering, University of Missouri, Columbia, MO 65211 USA; 5 Department of Physiology, Northwestern University, Chicago, IL 60611 USA; 6 Biomedical Engineering, College of Engineering, The University of Arizona, Tucson, AZ 85721 USA

**Keywords:** Neuroscience, Learning and memory, Fear conditioning

## Abstract

The positive or negative value (valence) of past experiences is normally integrated into neuronal circuits that encode episodic memories and plays an important role in guiding behavior. Here, we show, using mouse behavioral models, that glutamatergic afferents from the ventral tegmental area to the dorsal hippocampus (VTA→DH) signal negative valence to memory circuits, leading to the formation of fear-inducing context memories and to context-specific reinstatement of fear. To a lesser extent, these projections also contributed to opioid-induced place preference, suggesting a role in signaling positive valence as well, and thus a lack of dedicated polarity. Manipulations of VTA terminal activity were more effective in females and paralleled by sex differences in glutamatergic signaling. By prioritizing retrieval of negative and positive over neutral memories, the VTA→DH circuit can facilitate the selection of adaptive behaviors when current and past experiences are valence congruent.

## Abstract (web summary)

The neuronal pathway that signals the positive or negative value of memories is not well understood. Here, the authors report that an excitatory projection from the ventral tegmental area to the dorsal hippocampus carries the valence information, contributing, especially in females, to the recurrence of fear and to drug seeking behavior.

## Introduction

Positive or negative experiences usually lead to the formation of memories carrying similar valence^1^. Such memories (henceforth referred to as “positively or negatively valenced”) can impact threat avoidance and reward approach behaviors long after the original experience, not the least because their retrieval is often prioritized relative to neutral memories, especially in affective states that are congruent with their valence^2^. However, persistent affective memory bias can also have maladaptive consequences: negatively valenced memories contribute to anxiety disorders by triggering reinstatement of fear in situations that no longer predict danger^3^, while positively valenced memories contribute to addiction by triggering relapse of drug seeking despite an individual’s awareness of the drug’s life threatening consequences^4,5^. It is yet to be established whether sex differences in processing memory valence contribute to the heightened propensity of women to develop anxiety disorders^6^ and their higher susceptibility to drug-abuse relapse^7,8^.

The dorsal hippocampus (DH) plays a well-established role in the formation and retrieval of episodic memories^9^, that is, memories of personal experiences of events, the places in which they happened, and the times of their occurrence^10^. Our understanding of the cortical inputs to DH neurons, required for the formation of the sensory, spatial, and temporal components of episodic memories has significantly advanced in recent years^11–13^, particularly with the delineation of the roles of discrete entorhinal cortical projections to dentate gyrus (DG) granule cells^14,15^. Importantly, these cells also encode, in a flexible manner, the valence of memories of environmental contexts in which reinforcing events have taken place^16^. However, the circuits that signal valence to DG neurons have not been identified.

The activity of midbrain dopamine neurons in the ventral tegmental area (VTA) has long been implicated in signaling reward (both natural rewards and reward associated with drugs of abuse)^17,18^, as well as aversive outcomes^19,20^. However, a proposed role of DH-projecting VTA dopamine neurons in memory^21–23^, has recently been challenged because of the finding that dopaminergic projections from VTA to DH are scarce and that dopaminergic mechanisms of memory primarily involve projections from the locus coeruleus^24,25^. Apart from dopaminergic neuronal populations, VTA contains glutamatergic and GABAergic neurons^26–28^ that could contribute to DG granule cell activity^29^. We therefore sought to determine the extent of DH innervation provided by these projections and their role in driving behaviors based on valenced memories. We demonstrated a significant contribution of glutamatergic VTA→DH projections to the encoding of negative valence of context memories and to the reinstatement of fear after extinction. These projections also contributed to behavior guided by positively valenced memories, such as opioid-seeking following abstinence. The observed effects were especially strong in females, who showed higher density of VTA→DH terminals than males after reinstatement of fear or opioid-induced place preference, as well as faster decay of glutamate currents in response to activation of VTA→DH terminals. Together, these findings showed that excitatory VTA to DH projections provide a valence signal to memory circuits

## Results

### Characterization of VTA→DH projections

**Fig. 1:**
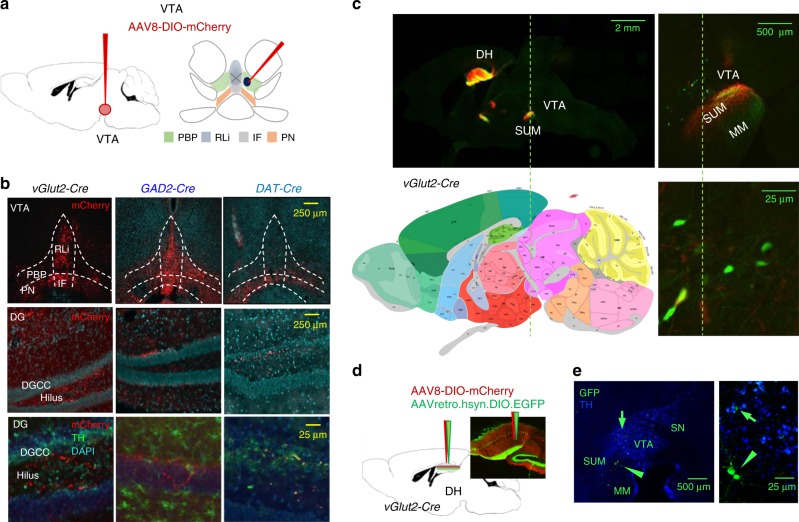
VTA→DH afferents mainly consists of glutamatergic projections. **a** Sagittal (left) and coronal (right) schematics of the neuroanatomical sites of Cre-dependent reporter virus injection in vGlut2-Cre (*n* = 3), GAD2-Cre (*n* = 3), and DAT-Cre (*n* = 3) mice. **b** Images showing virus infection (mCherry signals, red) in the VTA (top) and dentate gyrus of the hippocampus (middle). Bottom panel shows lack of co-localization of dentate gyrus mCherry signals with tyrosine hydroxylase (TH, green). In all sections DAPI (blue) is used as nuclear counterstain. Terminals from VTA were the most prominent in vGlut2-Cre mice. **c** Rostral VTA neurons localized above SUM showed prominent labeling with the retrograde tracer AAVretro.hsyn.DIO.EGFP (green) and were identified as DH-projecting VTA neurons. Different magnifications are shown top left and right. Bottom left: Localization was determined based on the Allen Brain Atlas. Credit for image: Allen Institute. **d** Schematics (left) and image (right) of injection of anterograde and retrograde viral tracers into DH (*n* = 4). **e** Retrogradely labeled DH-projecting VTA neurons were localized dorsal to SUM, as confirmed by TH immunostaining (blue). DG dentate gyrus, DGGC dentate gyrus granule cells, IF interfascicular nucleus, lm stratum lacunosum moleculare, MM mammillary body, PBP parabrachial pigmented nucleus, PN paranigral nucleus, RLi rostral linear nucleus of the raphe, SUM supramammilary body.

To trace and manipulate projections stemming from different glutamatergic, GABAergic, and dopaminergic neurons, we used Cre expressing mouse lines driven by the dopamine transporter (DAT-Cre), vesicular glutamate transporter 2 (vGlut2-Cre), or GABA decarboxylase 2 (GAD2-Cre) promoter, respectively. Injection of Cre-dependent AAV8-hM4(Gi)-mCherry viral vectors into the VTA of vGlut2-Cre, GAD2-Cre, and DAT-Cre mice revealed notable differences in mCherry immunostaining both in the VTA and in the DH. Consistent with existing evidence^28,30^, double immunofluorescence for tyrosine hydroxylase (TH) and mCherry in the VTA revealed predominant localization of TH in lateral VTA, in particular parabrachial pigmented nucleus, whereas mCherry signals were the strongest in medial VTA of vGlut2-Cre and GAD2-Cre mice, and in the paranigral nucleus of DAT-Cre mice (Fig. 1a, b). Whereas mCherry signals in DAT-Cre mice overlapped with TH, they only showed partial co-localization in medially and laterally labeled mCherry-positive neurons in vGlut2-Cre and GAD2-Cre mice (Supplementary Figs. 1–3). In DH, the strongest labeling was observed for vGlut2-positive terminals, particularly in the hilus of the DG, stratum lacunosum moleculare, and CA2/CA3 subfield. GAD2 terminals were mainly seen in the granule cell layer, DAT terminals in the hilus, and both in CA3 (Fig. 1b). Overall, terminals positive for DAT and GAD2 were scarce, and in many mice, DAT labeling was below detection level. Only DAT-positive terminals co-localized with TH (Fig. 1c), suggesting that vGlut2- and GAD2-positive terminals stem from TH-negative VTA neurons. Relative to the other notable glutamatergic VTA projections, including those to the lateral habenula, medial prefrontal cortex, and nucleus accumbens/caudate putamen (Supplementary Fig. 4), excitatory VTA→DH terminals were less abundant and detectable as a fine filamentous network in the hilus of the DG. Combined anterograde and retrograde tracing revealed that most DH-projecting VTA neurons are localized in the rostral VTA (Fig. 1c, d), and that their connectivity to DH is unidirectional.

In addition to VTA, the main neighboring area projecting to DH is the supramammilary nucleus (SUM). We therefore systematically controlled for virus spread in this area (Supplementary Fig. 5a), and apart from fibers of passage, we did not see infected neurons in SUM (Supplementary Fig. 5a–c). VTA terminals could also be differentiated from SUM terminals based on the DH labeling pattern because SUM injections resulted in much stronger DH labeling that was primarily confined to the DG cells rather than the hilus (Supplementary Fig. 6d).

### Chemogenetic inactivation of vGlut2-positive VTA→DH terminals impairs contextual fear conditioning

**Fig. 2:**
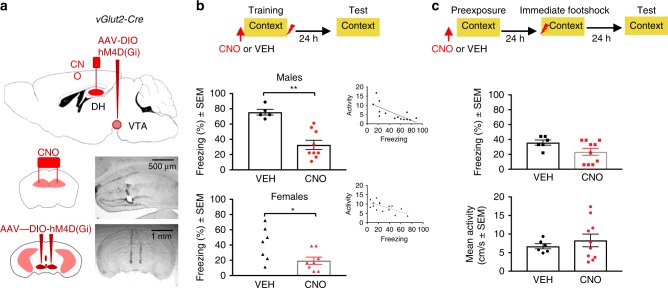
Chemogenetic inhibition of vGlut2 VTA→DH terminals impairs contextual fear conditioning in male and female mice. **a** Schematics and histological images demonstrating the cannula placement sites in DH (top and middle) and VTA injection sites of AAV8-DIO-hM4D(Gi) in VTA (top and bottom). **b** CNO significantly reduced freezing behavior during the memory test both in males [top, *t*_12_ = 4.886, **P = 0.004 CNO (*n* = 9) vs. VEH (*n* = 5) groups] and females [bottom, *t*_13_ = 2.367, *P = 0.0341, CNO (*n* = 7) vs. VEH (*n* = 8) groups] (right), as determined with two-tailed unpaired *t* test. Inset shows correlations between freezing scores and mean activity detected automatically by an infrared beam system (males, *r* = −0.7201, *P* = 0.0037, females, *r* = −0.6791, *P* = 0.0054). **c** CNO (*n* = 10) injected before context pre-exposure in the CPFE paradigm, did not affect freezing (*t*_15_ = 2.096, *P* = 0.0830, top) or locomotor activity (*t*_15_ = 0.6706, *P* = 0.5005, bottom) relative to VEH (*n* = 6), as determined with two-tailed unpaired *t* tests.

We recently demonstrated that local application of clozapine-N-oxide (CNO) in the brain effectively inhibits terminals arising from long-range DH-projecting neurons expressing AAV8-hM4(Gi)-mCherry^31^, and confirmed these observations in DH slices (Supplementary Fig. 6a). We therefore used this approach to determine whether VTA→DH projections contribute to contextual fear conditioning. Thirty minutes before training, mice expressing mCherry in VTA-originating terminals were injected with either vehicle (VEH) or CNO into DH (Fig. 2a). Injection of CNO did not affect activity to the context or shock at training (Supplementary Fig. 6b), but significantly impaired freezing behavior at test performed a day later. CNO was effective in both males and females (Fig. 2b). To determine whether the observed effect was specific for the encoding of negative contextual valence rather that the contextual representation, we also used a paradigm based on the context pre-exposure facilitation effect (CPFE)^32^, which allows delineating drug effects on context memory formation from context-shock associations^33^. In this paradigm, mice are pre-exposed to the to-be-conditioned context 1 day before exposure to an immediate shock, resulting in context-specific fear conditioning^34^ (Supplementary Fig. 6c, left). Injections of CNO before context pre-exposure were ineffective, demonstrating that silencing of VTA→DH did not affect the formation of context representations (Fig. 2c). Thus, the effects of CNO on context fear conditioning were most likely due to the association of context with the aversive footshock, resulting in a negatively valenced context memory.

### Silencing vGlut2-positive VTA→DH terminals abolishes reinstatement of fear

**Fig. 3:**
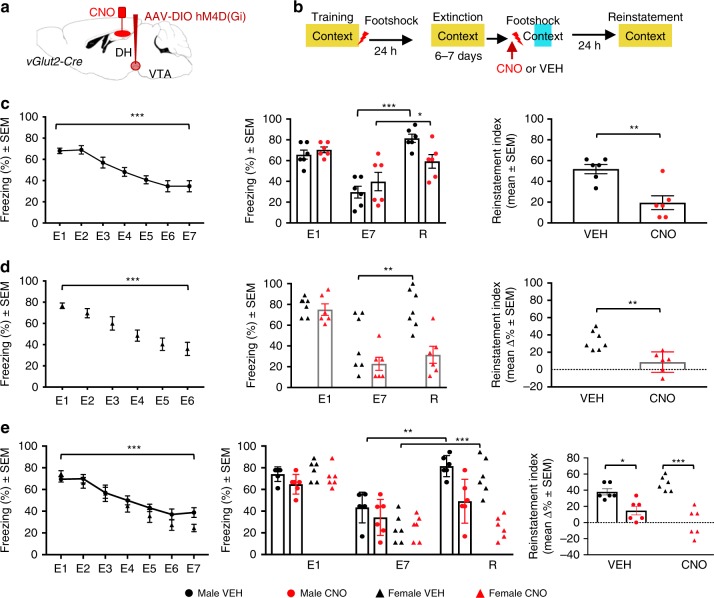
Silencing glutamatergic VTA→DH terminals prevents fear reinstatement. **a** Schematic of virus infusion into VTA and cannulation in DH. **b** Behavioral paradigm with context-footshock conditioning followed by exposure to context until extinction. Footshock reminder was presented in a novel context and mice were re-tested for fear reinstatement in the conditioning context. **c** Left, extinction in vGlut2-Cre male mice [RM one-way ANOVA, *F*_(2.841, 31.25)_ = 31.19, ***P < 0.0001]. Middle, comparison of freezing during the first context test (E1), last extinction test (E7), and reinstatement test in mice injected with VEH (*n* = 6) or CNO (*n* = 6) before reminder shock. Significant treatment × test interaction [two-way ANOVA, F_(2, 20)_ = 7.278, *P* < 0.0042] was found with stronger reinstatement in the VEH (***P < 0.0001, *n* = 6) vs. CNO (*P = 0.0175, *n* = 6) group. Right, CNO reduced the fear reinstatement index (two-tailed unpaired *t* test, *t*_10_ = 4.031, **P = 0.0024). **d** Left, extinction in vGlut2-Cre females [RM one-way ANOVA, *F*(3.034, 36.41) = 17.96, ***P < 0.0001]. Middle, treatment × test interaction after reminder shock (two-way ANOVA, *F*_(2, 22)_ = 8.316, *P* = 0.0020)), revealing significant reinstatement in the VEH (**P < 0.0002, *n* = 7) but not CNO group (*P* = 0.4890, *n* = 6)]. CNO reduced the reinstatement index (two-tailed unpaired *t* test, *t*_11_ = 3.936, **P = 0.0023 vs. VEH, right). **e** Males (*n* = 6) and females (*n* = 6) were directly compared for sex effects. Left, extinction [two-way ANOVA with Sex and Test, effect of Test, *F*_(6, 132)_ = 47.69, ***P < 0.0001]. Middle, in mice treated with VEH or CNO before shock reminder, three-way ANOVA revealed significant Sex [*F*_(1, 60)_ = 8.082, *P* = 0.0061] and treatment effects [*F*_(1, 60)_ = 32.50, *P* < 0.0001] on reinstatement, which was significant only in the VEH groups (males **P = 0.002, females ***P < 0.0001). Right, sex × treatment interaction [right, two-way ANOVA: *F*_(1, 20)_ = 5.839, *P* = 0.0254] and CNO-reduced fear reinstatement index (*F*_(1, 20)_ = 48.06, *P* < 0.0001, males *P = 0.0218, females ***P < 0.0001).

Fear conditioning followed by extinction typically results in the formation of negatively valenced (fear-inducing) and neutral (extinction) memories, with predominant retrieval of the neutral memory following sequential extinction trials^35,36^. To determine whether excitatory VTA→DH afferents bias retrieval towards fear-inducing context memories, we examined their potential contribution to fear reinstatement after extinction under valence congruent conditions (following reminder shock). vGlut2-Cre male mice were injected into VTA with Cre-dependent AAV8-hM4(Gi)-mCherry 5 weeks before cannula implantation, as illustrated in Fig. 3a). One week later, mice were trained in contextual fear conditioning, followed by extinction, shock reminder, and reinstatement test as described in the Methods and schematically outlined in Fig. 3b. The reminder shock specifically reinstated fear in the conditioning context without inducing fear conditioning to the context of immediate shock delivery (Supplementary Fig. 6c, right). One hour before the shock reminder, mice were injected with either VEH or CNO into DH. Following fear extinction (Fig. 3c, left) and shock reminder (Fig. 3c middle), vGlut2-Cre mice injected with VEH showed robust reinstatement of freezing. Compared with the last extinction test, reinstatement of freezing was reduced in CNO-treated mice and the reinstatement index was lower when compared with the VEH-treated group (Fig. 3c, right). We used the same chemogenetic approaches to silence SUM→DH terminals and examine the neuroanatomical specificity of the observed effects. In these experiments, CNO did not affect fear conditioning or reinstatement of fear after extinction (Supplementary Fig. 6d, e), which is consistent with the small contribution of SUM to hippocampus-dependent memory processes^37^ despite the abundance of SUM afferents in DH.

We subsequently performed a similar experiment in vGlut2-Cre females. Following extinction (Fig. 3d, left) and shock reminder (Fig. 3d, middle), the freezing response showed reinstatement in VEH-treated mice but was completely abolished in CNO-injected females, as revealed both by the lack of increase relative to the last extinction test (*P* = 0.4890) and between group differences of the reinstatement index (*P* < 0.01)(Fig. 3d, right). To directly examine the role of sex in the effects of VTA→DH terminals in fear extinction, we also investigated the effects of CNO in a separate experiment that included male and female mice (Fig. 3e). While replicating the main finding, this study also demonstrated a significant effect of sex, revealed by a sex × treatment interaction (*P* < 0.05).

### Optogenetic stimulation of vGlut2-positive VTA→DH projections trigger reinstatement of fear mediated by NMDA receptors

**Fig. 4:**
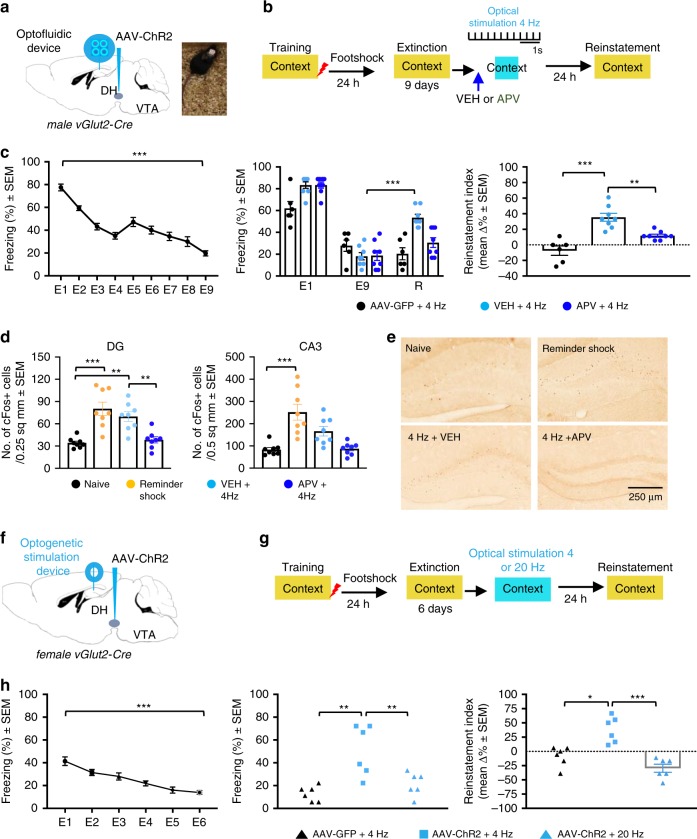
Optogenetic stimulation of vGlut2 VTA→DH terminals triggers fear reinstatement. **a** Virus infusion into VTA and implantation of optofluidic device in DH of vGlut2-Cre males. **b** After extinction, mice were injected with VEH (*n* = 8) or APV (*n* = 8) 20 min before receiving tonic 4 Hz stimulation. **c** Left, extinction [RM one-way ANOVA for all mice, *F*_(3.688, 77.45)_ = 43.19, ***P < 0.0001]. Middle, fear reinstatement after optogenetic stimulation. Two-way ANOVA revealed significant Stimulation effect [*F*_(2, 57)_ = 9.166, *P* = 0.0004], Test × Stimulation interaction [left, *F*_(4, 57)_ = 8.404, *P* < 0.0001], and reinstatement in the VEH-4 Hz group (***P < 0.0001). Right, increased reinstatement index in VEH but not APV or control virus (*n* = 6) groups [two-way ANOVA, *F*_(2, 19)_ = 21.96, *P* < 0.0001, ***P < 0.0001 VEH-4 Hz vs. AAV-GFP-4 Hz, **P = 0.0029 VEH- 4 Hz vs. APV-4 Hz]. **d** Upregulation of cFos in DG [*n* = 8/group, two-way ANOVA, *F*_(3, 28)_ = 14.05, *P* < 0.0001,, ***P < 0.0001 naïve vs. reminder shock, **P = 0.0016 naïve vs. VEH-4 Hz, **P = 0.0055 VEH-4 Hz vs. APV-4 Hz] and CA3 [two-way ANOVA, *F*_(3, 28)_ = 13.16, *P* < 0.0001, ***P < 0.0001 naïve vs. reminder shock]. **e** Illustration of cFos labeling. **f** Virus infusions and implantation of optostimulator devices in vGlut2-Cre females. **g** DH stimulation with 4 (*n* = 6) or 20 (*n* = 6) Hz for 1 min. **h** Left, extinction [RM one-way ANOVA for all mice, *F*_(2.866, 48.72)_ = 20.11, ***P < 0.0001]. Middle, fear reinstatement after stimulation of vGlut2 VTA**→**DH terminals with 4 (*n* = 6) but not 20 Hz (*n* = 6) in ChR2 but not GFP (*n* = 6) groups [one-way ANOVA, *F*_(2,15)_ = 11.27, *P* = 0.0010, **P = 0.0011 AAV-ChR2-4 Hz vs. AAV-GFP-4 Hz, **P = 0.0081 AAV-ChR2-4 Hz vs. AAV-ChR2-20 Hz). Right, reinstatement index [one-way ANOVA, *F*_((2, 15)_ = 19.32, *P* < 0.0001, *P = 0.023 AAV-ChR2-4 Hz vs. AAV-GFP-4 Hz, ***P < 0.0001 AAV-ChR2-4 Hz vs. AAV-ChR2-20 Hz].

We next sought to determine whether replacing the reminder shock with unilateral optogenetic stimulation of vGlut2-positive VTA→DH projections would affect fear reinstatement. Mice were infused with AAV8-Ef1a-DIO hChR2-EYFP or control virus AAV-EF1a-DIO-EGFP and 3 weeks later implanted with optofluidic devices (Fig. 4a). Based on pilot data involving different protocols (including 20 Hz stimulation shown below), we injected the mice with VEH or APV, and 20 min later applied tonic, 4 Hz blue light stimulation over 3 min (Fig. 4b). The unilateral design enabled to determine the effects of APV on optogenetic stimulation without interfering with fear reinstatement independently (Supplementary Fig. 7a). After fear conditioning, extinction (Fig. 4c, left), and 4 Hz tonic optogenetic stimulation in the presence of VEH or APV, vGlut2-Cre mice were placed in the conditioning context to record freezing behavior. Mice expressing hChR2 showed significant reinstatement of freezing after optogenetic stimulation performed in the presence of VEH (*P* < 0.001), whereas mice expressing a control virus and mice expressing hChR2 but stimulated in the presence of APV did not show reinstatement (*P* = 0.4947). This was revealed both by two-way ANOVA of freezing behavior (Fig. 4c, middle) and by between group comparison of the reinstatement index (Fig. 4c, right**)**. Importantly, unilateral optogenetic stimulation itself was devoid of aversive reinforcing properties, as indicated by lack of freezing behavior after pairing of a conditioning context with optogenetic stimulation instead of footshock (Supplementary Fig. 7b).

To determine how optogenetic stimulation affected neuronal activity within the DH, we quantified cFos responses 1 h after stimulation or reminder shock. Exposure to a reminder shock resulted in a significant upregulation of cFos-positive neurons in the CA3 subfield and in the DG (Fig. 4d, e, Supplementary Fig. 8). When compared with a reminder shock, optogenetic 4 Hz stimulation did not significantly induce cFos activity in CA3 (*P* = 0.4113), however levels in DG were similar to those induced by the shock reminder and significantly higher than in naïve (*P* < 0.01) controls and APV-injected mice (*P* < 0.01).

Similar to males, females implanted with devices for optogenetic stimulation end expressing ChR2in their VTA→DH terminals (Fig. 4f), were presented with tonic 4 Hz light pulses in the DH instead of exposure to shock reminder (Fig. 4g). Addition of the 20 Hz stimulation protocol served to further control for the specificity of stimulation effects. Following fear conditioning, extinction (Fig. 4h, left), and optogenetic stimulation, females stimulated with 4 Hz but not 20 Hz showed significant reinstatement of freezing behavior (*P* < 0.001, Fig. 4h, middle and right). Thus, in males and in females, activity of vGlut2-positive VTA→DH terminals was both necessary and sufficient to trigger fear reinstatement.

### Chemogenetic inactivation of vGlut2-positive VTA→DH terminals also abolishes reinstatement of morphine-induced conditioned place preference (M-CPP)

**Fig. 5:**
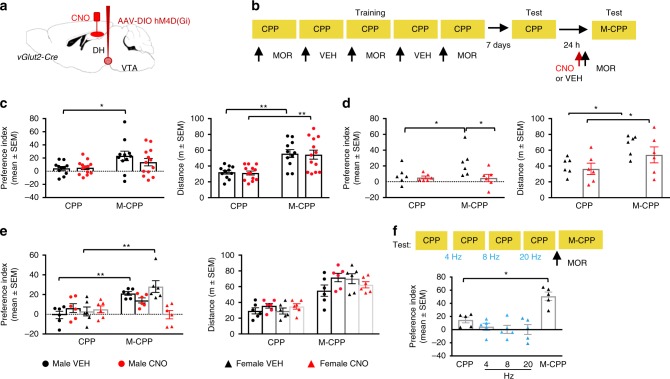
Silencing VTA→DH terminals impairs reinstatement of M-CPP. **a** Virus infusion into VTA and cannula implantation in DH. **b** Mice were trained in M-CPP triggered by a priming dose of morphine. One hour before M-CPP, VEH (*n* = 11) or CNO (*n* = 13) were injected into DH. **c** In vGlut2-Cre males, M-CPP was detected in VEH but not CNO group [left, two-way ANOVA, Test effect *F*_(1, 44)_ = 7.909, *P* = 0.0073, *P = 0.0498 VEH-CPP vs. VEH-M-CPP, *P* = 0.5198 CNO-CPP vs. CNO-M-CPP], but there was no group effect (left, two-tailed unpaired *t* test, *t*_22_ = 1.054, *P* = 0.3035). Enhanced of locomotion was similar in both groups [right, two-way ANOVA, *F*_(1, 44)_ = 30.28, *P* < 0.0001, **P = 0.0027, VEH CPP vs. M-CPP, **P = 0.0012, CNO CPP vs. M-CPP]. **d** In vGlut2-Cre females, CNO (*n* = 6) preve*n*ted M-CPP, as revealed by within subject [two-way ANOVA, *F*_(1, 20)_ = 4.3, *P* = 0.0497, *P = 0.0383 VEH CPP vs. M-CPP), and group effects [unpaired two-tailed *t*-test, *t*_10_ = 2.25, *P = 0.0482 vs. VEH (*n* = 6)]. Locomotor activity was not affected (two-way ANOVA, *F*_(1, 10)_ = 2.26, *P* = 0.1484), showing increases in both groups (*P < 0.0252 VEH CPP vs. M-CPP, *P = 0.0328 CNO CPP vs. M-CPP). **e** Sex effects in males (*n* = 6) and females (*n* = 6). Three-way ANOVA demonstrated sex × treatment interaction [*F*_(1, 40)_ = 5.276, *P* = 0.0269], with M-CP*P* in VEH groups only (**P = 0.0099 males and **P = 0.0018 females), and similar locomotion [*F*_(1, 40)_ = 3.366, *P* = 0.0740]. **f** Females (*n* = 5) received optoge*n*etic stimulation on consecutive days followed by a priming dose of morphine. RM one-way ANOVA revealed significant effect of treatment, *F*(2.167, 8.66) = 16.36, *P* = 0.0009, however, while morphine-induced place preference (M-CPP vs. CPP, *P = 0.0465), stimulations were ineffective (4 Hz vs. CPP *P* = 0.5109, 8 Hz vs. CP*P P* = 0.2307, 20 Hz vs. C*P*P *P* = 0.4556).

We next examined whether the role of VTA→DH projections extends to the retrieval of positively valenced memories under valence congruent conditions. To ensure that behavior was primarily driven by the rewarding properties of morphine rather than contextual cues, we used a place preference paradigm paradigm that results in subthreshold CPP (about 10%, not significant from chance) but robust (about 30% or more) M-CPP accompanied by increased total locomotion. We also used a priming dose of morphine that was ineffective in the absence of previously conditioned M-CPP memory (*P* = 0.9848, Supplementary Fig. 9a, b). Also, this paradigm is not sensitive to stress-induced CPP (S-CPP) (Supplementary Fig. 9c, d), which rules out a confounding effect of negatively valenced information as potential trigger of CPP. Surgeries and virus infusions were performed as described above, and 5 weeks later mice were trained in M- CPP as described in the Methods and schematically shown in Fig. 5a, b. vGlut2-Cre mice injected with vehicle displayed significant M-CPP (main effect of test: *F* (1, 22) = 10.98, *P* = 0.0032, post hoc comparison with off drug CPP, *P* = 0.0099), an effect that was not found in vGlut2-Cre mice injected with CNO (post hoc comparison with off drug CPP, *P* = 0.2727) (Fig. 5c). However, there were no significant group differences of M-CPP [vGlut2-Cre: *F* (1, 22) = 0.5639, *P* = 0.4607] nor changes of morphine-induced total activity in the CPP apparatus [vGlut2-Cre: *F* (1, 22) = 0.04847, *P* = 0.8278] relative to controls. In contrast to the partial effect observed in males, chemogenetic silencing of vGlut2-positive VTA→DH terminals completely abolished M-CPP (*P* = 0.9916) in females, indicating the lack of place preference, without interfering with total locomotion (Fig. 5d) or with CPP in the absence of morphine (Supplementary Fig. 10a). This sex difference was replicated in a separate experiment performed with both males and females (Fig. 5e), and was revealed, using three-way ANOVA) as significant sex × treatment interaction (*P* < 0.05). We next examined, using vGlut2-Cre females, whether M-CPP can be induced using optogenetic stimulation of VTA→DH terminals. However, three different protocols, involving stimulation with 4 Hz, 8 Hz, or 20 Hz between two off drug tests (Fig. 5f), or during CPP or M-CPP proved ineffective (Supplementary Fig. 10b, c). These results showed a complex contribution of VTA→DH projections to behavior based on positively valenced memories, with greater overall involvement in females than in males. However, even though activation of these projections was both necessary and sufficient for fear reinstatement, it was necessary but not sufficient for M-CPP.

### Chemogenetic inactivation of GAD2-positive VTA→DH terminals does not affect fear reinstatement or M-CPP

**Fig. 6:**
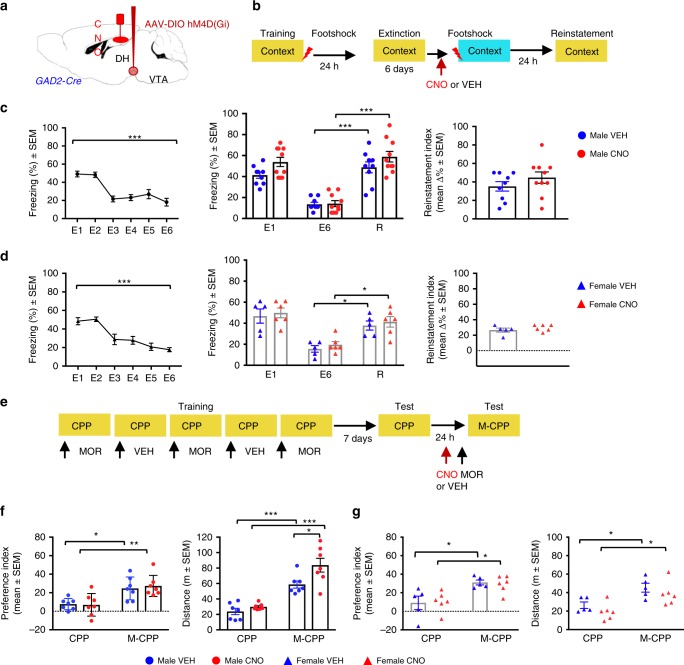
Silencing VTA→DH neurons in male and female GAD2-Cre did not affect fear reinstatement or M-CPP. **a** Virus infusion into VTA and cannulation in DH. **b** Fear reinstatement procedure. **c** Left, extinction in GAD2-Cre males [RM one-way ANOVA, *F*_(3.049, 54.88)_ = 18.5, *P* < 0.0001]. Middle, reinstatement of freezing behavior in VEH (*n* = 9, ***P < 0.0001) and CNO (*n* = 10, ***P < 0.0001) groups without treatment × test interaction [two-way ANOVA, *F*_(2, 51)_ = 1.259, *P* = 0.2925]. Right, CNO did not affect the fear reinstatement index (right, two-tailed unpaired *t* test, *t*_17_ = 1.178, *P* = 0.2550). **d** Extinction in GAD2-Cre females [left, *F*_(2.686, 26.86)_ = 15.17, *P* < 0.0001]. CNO did not affect fear reinstatement [middle, two-way ANOVA revealed effects for Test: *F*_(2,27)_ = 19.95, *P* < 0.0001, VEH (*n* = 5) *P = 0.0339, CNO (*n* = 6) *P = 0.0189 but not treatment *F*_(1, 9)_ = 0.2262, *P* = 0.3657 or treatment × test interaction: *F*_(2, 27)_ = 0.0, *P* = 0.9956] or the reinstatement index (right, two-tailed unpaired *t* test, *t*_9_ = 0.9585, *P* = 0.3629). **e** M-CPP procedure. **f** Chemogenetic inactivation of GAD2 terminals in males did not affect M-CPP [two-way ANOVA *F*_(1, 24)_ = 0.05301, *P* = 0.8199, VEH (*n* = 7) *P = 0.0143, CNO (*n* = 7) **P = 0.0035] but enha*n*ced morphine-induced locomotion [two-way ANOVA treatment effect *F*_(1, 24)_ = 8.048, *P* = 0.0091, VEH **P = 0.0008, CNO ****P < 0.0001, two-tailed unpaired *t*-test *t*_12_ = 2.483, *P = 0.0288]. **g** In GAD2-Cre females, chemogenetic silencing did not affect M-CPP [left, two-way ANOVA, *F*_(1, 18)_ 0.001841, *P* = 0.9662, two-tailed unpaired *t* test *t*_9_ = 0.01115, *P* = 0.9921, CNO (*n* = 6) vs. VEH (*n* = 5)]. Locomotor activity was also not affected [right, two-way ANOVA *F*_(1, 18)_ = 2.381, *P* = 0.1402, two-tailed unpaired *t* test *t*_9_ = 0.5042, *P* = 0.6642]. Significant increases in M-CPP (VEH* *P* = 0.0255, CNO *P = 0.0385) and activity (VEH *P = 0.0433, CNO *P = 0.0213) were found in all groups.

We next examined whether the effects observed with chemogenetic approaches were restricted to vGlut2-positive VTA→DH terminals or also occur after silencing of GAD2-containing projections. We performed similar experiments as described above with GAD2-Cre male and female mice (Fig. 6), however the only detectable effect was on total activity in the M-CPP paradigm in males (Fig. 6f) but not in females (Fig. 6g). Overall, freezing after fear conditioning was lower in this mouse strain relative to vGlut2-Cre mice (~40–50% vs. 60–70%). We therefore performed an additional experiment with GAD2-Cre male mice using three shock presentations that resulted in similar freezing level as shown for vGlut2-Cre males (Fig. 3c). However, CNO remained ineffective (Supplementary Fig. 11). Together, the findings indicated a lack of significant contribution of GAD2-Cre VTA→DH afferents to the retrieval of fear-inducing or M-CPP memories.

### vGlut2-positive VTA→DH terminals are more abundant in females and show sex-specific differences in glutamate current kinetics

**Fig. 7:**
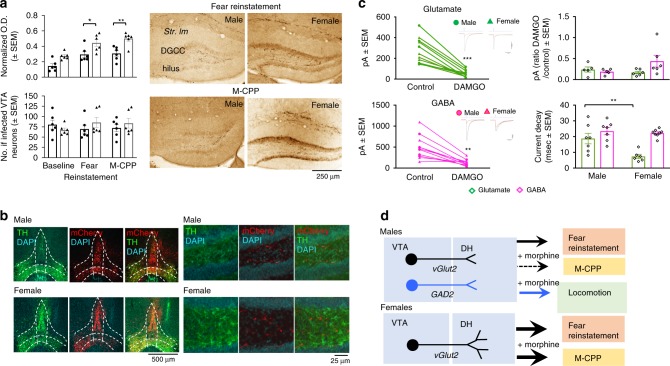
Sex differences in the density of VTA→DH vGlut2 terminals and their effects on excitatory transmission. **a** Density of vGlut2-positive VTA-DH terminals in DG under baseline conditions (*n* = 6 males, *n* = 6 females), after fear reinstatement (*n* = 6 males, *n* = 6 females), and after M-CPP (*n* = 6 males, *n* = 6 females) revealed significant sex differences (top left, two-way ANOVA *F*_1,30_ = 34.71, *P* < 0.0001), with females showing significantly higher density after reinstatement (*P = 0.0216) and after M-CPP (**P < 0.0040]. In the same mice, the number of VTA-infected neurons was similar [bottom left, Sex: *F*_(1, 30)_ = 0.3486, *P* = 0.5593, Group: *F*_(2, 30)_ = 0.1023, *P* = 0.9031]. These differences are illustrated with mCherry images of DG (right). **b** In either sex, vGlut2 VTA DH terminals did not show detectable co-localization with tyrosine hydroxylase (TH). **c** Optogenetic stimulation of VTA terminals triggered glutamate and GABA currents whose amplitude was significantly decreased in the presence of DAMGO [glutamate [*n* = 11 (five males and six females): *F*_(1, 9)_ = 48.24, ***P < 0.0001; GABA (*n* = 11): *F*_(1, 9)_ = 24.93, **P = 0.0007]. A significant effect of DAMGO was also found with nested ANOVA factoring for data from individual mice [*F*(_1,21)_ = 15.40, *P* = 0.017]. The amplitude ratios of these currents did not differ between sexes [two-way ANOVA, *F*_(1, 18)_ = 1.118, *P* = 0.3044], however, there were significant effects of sex [*F*_(1, 24)_ = 8.302, *P* = 0.0082] and current type [*F*_(1, 24)_ = 19.98, *P* = 0.0002] on the current decay. Post hoc tests revealed that the decay of glutamate currents was significantly faster in females (**P = 0.0033) than in males, whereas GABA currents had similar decay rate (*P* = 0.8949). Nested ANOVA including individual mice as a variable also revealed a significant effect of sex on current decay [*F*(_1,21)_ = 9.2065436, *P* = 0.038]. **d** Summary of the effects of glutamatergic and GABAergic VTA**→**DH terminals in fear reinstatement and M-CPP. The thickness of the arrow reflects the strength of the effect.

To gain first insight into the stronger involvement of vGlut2-positive VTA→DH projections in behavioral regulation in females, we compared the density of their DH terminals in males and females and found a significantly denser innervation of the stratum lacunosum moleculare, CA3 and especially in the DG (*F* (1, 24) = 20.89, *P* < 0.001) of females (Supplementary Fig. 12). These differences were significant after fear reinstatement and M-CPP (Fig. 7a). Two-way ANOVA revealed significant effects of sex [*F* (1, 30) = 30.61, *P* < 0.001] and Group [*F* (2,30) = 17.75, *P* < 0.001]. This was not due to the greater labeling of VTA neurons in females because, when compared with males, females had similar numbers of VTA neurons expressing mCherry [*F* (2,30) = 0.3486, *P* < 0.5593] (Fig. 7a, Supplementary Fig. 12). In addition, the effect was not general for all VTA projections, as revealed by the lack of sex-specific difference of VTA terminals in the lateral habenula [*F* (1,30) = 0.009854, *P* < 0.9216, mean ± SEM: baseline males 0.946 ± 0.015, females 0.928 ± 0.02, fear reinstatement males 0.930 ± 0.22, females 0.955 ± 0.016, M-CPP males 0.924 ± 0.025, females 0.923 ± 0.034; source data provided with Fig. 7a]. We also explored whether females might receive terminals co-expressing vGlut2 and TH that could contribute to M-CPP by engaging dopamine signaling. However, lack of co-localization in DH ruled out this hypothesis (Fig. 7b).

We next compared the cellular effects of vGlut2-containing VTA→DH projections in males and females. Given that optogenetic stimulation of these projections triggers dual release of glutamate and GABA^29^, we performed the experiments under conditions that allow recording glutamate and GABA-induced currents from DH slices collected 4 weeks after infusion of AAV2-DIO-ChR2 into the VTA. As expected, optogenetic stimulation triggered both types of currents with similar amplitudes in males and females (Fig. 7c, top), but with significantly faster decay of the glutamate current in females (Fig. 7c, bottom). These findings suggest that in addition to the greater abundance of terminals innervating granule cells, differences in the biophysical properties of the glutamate current may also contribute to the stronger involvement of glutamatergic VTA→DH projections in females. We also recorded in the presence of the µ opioid receptor (MOP) agonist DAMGO, to examine the functioning of VTA→DH projections under conditions relevant for M-CPP. In the presence of DAMGO, all current amplitudes were significantly reduced in both sexes (changes of individual amplitudes and representative traces are shown in Fig. 7c, middle and right, respectively). Thus, differences in glutamatergic signaling might contribute to the sex-specific roles of vGlut2-positive VTA→DH projections in the retrieval of valenced memories.

## Discussion

In this comparative study of the distribution and function of VTA→DH projections, we identified a glutamatergic VTA→DH circuit that impacts the activity of DG cells and provides key input for valence signals to the DH. Surprisingly, this circuit appears to drive both fear reinstatement and morphine seeking, especially in females. These findings provide a long-sought link between VTA and DH that drives motivated behavior rooted in negatively, and, to a lesser extent, positively valenced memories.

Although studies that have focused on the most abundant VTA projections often fail to report the presence of VTA→DH efferents^38,39^, the identification of rostral VTA neurons as the primary source of DH efferents in this work is consistent with tracing studies focusing on hippocampal VTA projections^29,40,41^. Despite their sparsity relative to other VTA projections, vGlut2-containing VTA→DH terminals proved to be of high functional significance for the formation and retrieval of negatively valenced relative to neutral context memories. The chemogenetic and optogenetic approaches used throughout the experiments were selected based on their superior efficacy in silencing (chemogenetic) and activating (optogenetic) terminals relative to cell bodies^42^. Using these approaches, we demonstrated that these afferents are both necessary and sufficient for the reinstatement of freezing even after extensive extinction. It was recently demonstrated that optogenetic 4 Hz stimulation of the prefrontal cortex coordinates activity of prefrontal-amygdala circuits and elicits freezing behavior^43^. A similar mechanism, synchronization of hippocampus-amygdala circuits at 4 Hz, which is known to contribute to freezing behavior^44^, might account for fear reinstatement induced by 4 Hz stimulation of VTA→DH afferents in our study. We did not observe effects with 20 Hz stimulation that was previously effective in inducing reinstatement of avoidance behavior^16^, which could be due the fact that we stimulated terminals of long-range projections rather than DG cells. The sufficiency of our protocol to trigger fear reinstatement enabled us to demonstrate, by using wireless optofluidic devices, that NMDA receptors are the mediators of this effect. This is in agreement with previous work^45,46^, showing that, as in fear conditioning^47^, glutamatergic-induced plasticity mediates fear reinstatement, and provides new evidence that such plasticity is facilitated by excitatory VTA→DH afferents.

Contrary to other VTA projections (e.g., to the nucleus accumbens or lateral habenula)^48,49^, activation of vGlut2 VTA→DH projections under the conditions used in our study did not induce behavioral changes consistent with the primary aversive or appetitive reinforcement of footshock and priming dose of morphine, respectively. This was concluded from the findings that chemogenetic silencing or optogenetic stimulation of vGlut2 VTA→DH terminals did not affect locomotion and shock responses during fear conditioning and could not substitute for the shock input. Although we cannot rule out the possibility that reinforcing effects might occur with stronger stimulation or with other behaviors, our findings show that negative valence signals provided by VTA→DH afferents are not sufficient to drive unconditional freezing behavior, and suggest that integration of VTA signals with input from other afferents by may be required (e.g., sensory information from the entorhinal cortex for context/place representations^15^) for fear conditioning. Nevertheless, stimulation of these afferents was sufficient for reinstatement of freezing behavior, suggesting that they provided negative valence-congruent signals facilitating retrieval of fear-inducing over neutral memories. VTA→DH projections also contributed to M-CPP although their stimulation could not substitute for morphine, nor affected locomotion or side preference in M-CPP. This suggests that in addition to VTA→DH input, DG neurons might need to integrate other inputs, as discussed above, or act in synergy with primary reinforcement circuits, such as amygdala and nucleus accumbens, to induce positive valence congruent signals needed for retrieval of opioid-related memories. The former possibility is supported by observations that direct activation of DG cells, simulating responses to multiple inputs, can induce approach toward natural rewards^16^.

Unlike the typically differentiated roles of glutamatergic, dopaminergic, and GABAergic projections in aversion and reward (e.g., to the nucleus accumbens, lateral habenula, and medial prefrontal cortex)^28^, such differentiation was less evident in vGlut2-positive VTA→DH projections. Clearly, under drug-free conditions, these projections contributed to fear-motivated behavior. However, under morphine, the same projections contributed, especially in females, to the preference for a context previously associated with reward. Given the multiplexed neurochemical composition of VTA neurons^50^, it is possible that there is a further, so far unknown, differentiation within vGlut2-positive VTA→DH projections that signal valence information. In view of recent findings, demonstrating that the same glutamatergic VTA neurons respond to aversion and reward^51^, it is more likely that the activity of these projections could be interpreted state-dependently under conditions that trigger aversion (e.g., shock reminder or 4 Hz stimulation) versus reward (e.g., morphine). Accordingly, in response to VTA terminal activation, DG cell current properties differed substantially depending on mu opioid receptor activation, raising the interesting possibility that DG cell current amplitudes might be related to the polarity of valence (e.g., high amplitude currents signaling negative, and low amplitude currents signaling positive memory valence). This is consistent with findings showing that the same excitatory VTA neurons respond with increased firing to aversive and decreased firing to rewarding stimuli^51^. This possibility is also in line with emerging support for state-dependent rather than dedicated processing of positive and negative valence^52,53^.

The abundance of vGlut2-positive terminals and kinetics of glutamate currents might have contributed to the stronger impact of excitatory VTA→DH projections in fear reinstatement and M-CPP in females than in males. Interestingly, a similar, female-specific increase of vGlut2-positive terminals was found in the nucleus accumbens in response to stress^54^, however, it is not known whether this increase was restricted to VTA afferents or occurs more generally as a reflection of enhanced stress sensitivity in females. Given that the total number of VTA neurons was similar across conditions, it is likely that the causes for these differences are to be found in mechanisms underlying activity-dependent axonal branching or synaptic connectivity of VTA→DH terminals^55–57^. In addition, the observed sex differences could be due, at least in part, to the faster decay of glutamatergic (but not GABAergic) currents in females than in males, which might indicate the engagement of different glutamate receptor types or NMDAR subunits in driving fear reinstatement and M-CPP in females and males^58,59^.

It was recently suggested that GAD2-Cre and vGlut2-positive VTA→DH terminals similarly regulate the activity of DG granule cells through GABA and glutamate-co-release^29,60^. However, we found that these projections markedly differ in abundance, localization, and function, what could account for the profound impact of chemogenetic inactivation of vGlut2-positive, but not GAD2-positive VTA→DH projections, on fear reinstatement and M-CPP. Replication of these findings with additional mouse lines, such as vGat-Cre^61^, is yet to be performed to strengthen this view. The only behavioral phenotype found by silencing GABAergic VTA afferents in DH was a male-specific contribution to morphine-induced locomotion. Because place preference and locomotion are viewed as competing behaviors^62^, the role of the inhibitory VTA→DH projections in constraining locomotion could synergize with the enhancement of place preference by excitatory VTA→DH projections, and thus coordinate M-CPP in males.

Overall, our findings show that excitatory, non-dopaminergic VTA neurons signal negative and positive valence to DH memory circuits. This could prioritize the retrieval of negative and positive over neutral memories, but also ensure flexible interpretation of contextual stimuli with changing experiences. While such circuit mechanisms have an important adaptive value in organizing threat avoidance and reward approach behaviors under valence congruent conditions, it is likely that excessive bottom-up control of DH by VTA could also drive persistent reinstatement of fear and opioid seeking, which, in humans, might contribute to the development of anxiety disorders and opioid addiction. Moreover, the observed sex differences might prove relevant to the heightened vulnerability of females to these disorders.

## Methods

### Mice

We performed the experiments using male and female C57BL/6J mice, vGlut2-Cre, GAD2-Cre, and DAT-Cre mice. Wild type C57BL/6J mice were purchased from Harlan, Indianapolis, IN). All other mouse lines were obtained from the Jackson Laboratory (Bar Harbor, ME). The vGlut2-Cre knockin mice, also known as Slc17a6tm2(cre) and Lowl or VGlut2-ires-Cre, express Cre recombinase in excitatory glutamatergic neuron cell bodies containing vGlut2 (ref.^63)^. The GAD2-Cre mice (Gad2^tm2(cre)Zjh^/J), developed and characterized by Taniguchi et al.^64^, express Cre in GABAergic neurons, whereas DAT-Cre mice [B6.SJL-Slc6a3tm1.1(cre)Bckmn/J], developed and characterized by Backman et al.^65^, express Cre in dopaminergic neurons containing the DAT.

Heterozygous mice were backcrossed with wild-type C57BL/6J for six generations in our facility to achieve offspring with a genetic identity which is closer to the C57BL/6J strain. The colony was subsequently expanded by homozygous breeding and confirmed by genotyping using the primers and protocols posted on the Jackson Laboratory website (vGlut2: common, CGG TAC CAC CAA ATC TTA CGG, mutant reverse ATC GAC CGG TAA TGC AGG CAA, wild type reverse CAT GGT CTG TTT TGA ATT CAG; GAD2-Cre: common AAC AGT TTG ATG AGT GAG GTG A, mutant forward CAC TGC ATT CTA GTT GTG GTT TG, wild type forward TCG TTG CAC TGA CGT GTT CT; DAT-Cre: commonTGG CTG TTG GTG TAA AGT GG, mutant reverse CCA AAA GAC GGC AAT ATG GT, wild type reverse GGA CAG GGA CAT GGT TGA CT. Typically, we obtained 4–6 l/breeding cycle with 5–8 mice/l with similar distribution of males and females. All mice were 8 weeks of age at the beginning of the experiments. The mice were maintained under standard housing conditions (12/12 h light–dark cycle with lights on at 7 a.m., temperature 20–22 °C, humidity 30–60%) in our satellite behavioral facility. All behavioral experiments were performed between 9 a.m. and 5 p.m. Randomization was performed by assigning similar numbers of littermates to the different treatment conditions. The behavioral experiments were performed by two experimenters of which one was blind to genotypes and drug treatments. All animal procedures used in this study were approved by the Northwestern University IACUC and complied with federal regulations set forth by the National Institutes of Health.

### Contextual fear conditioning, extinction, and reinstatement

Contextual fear conditioning was performed in an automated system (TSE Systems)^66^. Mice were exposed for 3 min to a novel context, followed by a footshock (2 s, 0.7 mA, constant current), and tested for memory retrieval 24 h later by returning them to the conditioning context for 3 min. Freezing was scored every 10 s during context exposures and expressed as a percentage of the total number of observations during which the mice were motionless. Activity was recorded automatically by an infrared beam system and expressed as cm/s. Extinction trials consisted of daily exposures of mice to the conditioning context for 3 min without footshock. Criterion for successful extinction of fear was significant decrease of average group freezing during three successive exposures to the context when compared with the first extinction trial, as determined by repeated measures ANOVA. After the last extinction trial, the mice were split into experimental and control groups so that the mean % freezing/group would be similar while randomizing the distribution of littermates to prevent litter effects. Fear reinstatement was induced by placing the mice to an immediate 2-s 0.7 mA footshock in a novel context. Mice were returned to the conditioning context or novel context the following day and freezing was determined during re-exposure to the conditioning context. This procedure does not induce fear conditioning but serves as a reliable trigger for fear reinstatement^67^. The difference between the percentage of time spent freezing during reinstatement and the percentage of time spent freezing during the last extinction trial (Δ%) served as a reinstatement index.

*CPFE Paradigm*. We performed the CPFE paradigm as described previously^33^. Prior to pre-exposure, mice were handled and exposed to the transport cages for 5 min/day for 2 days. During pre-exposure mice were transported to the conditioning context and allowed to explore for 10 min (pre-exposed group). On day 2, all mice were transported to the conditioning chambers, three later administered a 2-s, 0.7 mA footshock, and remained in the context for 60 s. On day 3 mice were transported to the conditioning context, or, in control experiments different context, and freezing was assessed for 1 s every 5 s over 3 min.

### Morphine-induced conditioned place preference (M-CPP)

CPP took place in an apparatus consisting of three chambers: two conditioning chambers each with distinct tactile, visual, and olfactory cues and a small connecting brightly lit neutral chamber. White background noise was delivered to block out any possible extraneous acoustic cues. On the first day (preconditioning phase) mice were placed into the neutral chamber and then allowed to freely explore all three chambers for 15 min. Over the next 5 days, mice received either morphine (5 mg/kg in 200 µl subcutaneously (s.c.) prior to a 30-min confinement in one of the chambers (days 1, 3, and 5) or 200 µl of saline prior to confinement to the other chamber (days 2 and 4). One week later, mice were first tested off drug to determine conditioned place preference (CPP), and 24 h later after treatment with a priming dose of morphine (2 mg/kg s.c.) to determine morphine-induced CPP (M-CPP). In a control experiment we delivered 15 inescapable 1-s footshocks, 0.5 mA constant current, over 15 min to determine whether stress can induce CPP (S-CPP) in this paradigm.

During tests, mice were allowed to freely explore all chambers for 15 min and time spent in the chambers was measured using Any-maze tracking software. For each animal, the less preferred compartment during the preconditioning phase was used as the morphine-paired assigned side. Mice showing over 80% preference for one chamber during preconditioning were excluded (a total of 5). Place preference was indexed by the difference between the percentage of time in the morphine-paired chamber at test and the percentage of time in the same chamber during preconditioning.

### Stereotaxic surgeries and infusions of viral vectors and drugs

Mice were anesthetized with 1.2% tribromoethanol (vol/vol, Avertin) for viral vector intracranial infusion and cannula implantation. The viral vector carrying a construct coding for the Cre-independent inhibitory DREADD (AAV8-hSyn-HA-hM4D(Gi)-mCherry, Addgene 44362), Cre-dependent inhibitory DREADD (AAV8-hSyn-DIO-hM4D(Gi)-mCherry, Addgene 50475), AAV8-Ef1a-DIO hChR2(E123T/T159C)-EYFP, Addgene 33509), or AAV-EF1a-DIO-EGFP (Vector Biolabs VB2088) was bilaterally infused into the VTA (3.4 mm posterior, ±0.5 mm lateral, 4.3 mm ventral to bregma) or SUM (2.8 mm posterior, ±0.5 mm lateral, 4.5 mm ventral to bregma). Infusions were performed using an automatic microsyringe pump controller (Micro4-WPI) connected to a Hamilton microsyringe. The viral vectors were infused in a volume of 0.35 μL per site, at titer ≥3 × 10^12^ vg/mL, over 2 min, and syringes were left in place for 5 min prior to removal to allow for virus diffusion. For retrograde tracing, we used retrograde particles produced from AAV-hSyn-DIO-EGFP allowing from Cre-dependent enhanced green fluorescent protein (EGFP) expression (Adgene, 50457-AAVrg, at titer ≥7 × 10^12^ vg/mL). The vector was infused into the dorsal DG (1.8 mm posterior, ±1.0 mm lateral, 2.5 mm ventral to bregma, 350 µl, unilaterally).

Bilateral 26 gauge guide cannulas (Plastics One) were placed in DH (1.8 mm posterior, ±1.0 mm lateral, 2.25 mm ventral to bregma). Mice were allowed 6 weeks for virus expression prior to behavioral testing. CNO (Sigma; 0.3 μg/mL; 0.20 μL per side, at a rate of 0.5 μL/min) was infused through the cannulas 60 min prior to context pre-exposure, fear reinstatement, or reinstatement of M- CPP. After the completion of behavioral testing, all brains were collected and cannula placements and virus spread were confirmed by immunohistochemical analysis of mCherry or EGFP signals. The density of mCherry signals and number of cFos-positive nuclei were quantified using Image J_68._ For quantification of cFos nuclei in the DG the entire area was outlined, and counts were performed in the defined area. Constant threshold was used to distinguish nuclei >5 μm and with the intensity above 0.47 arbitrary units. Counts were then normalized to area. For densitometric analyses, two 100 um^2^ counting frames were defined at the same coronal level as DG analyses −1.8 mm posterior to the bregma. Background was the same for all sections because the primary antibodies did not generate any nonspecific signals.

### Implantation of devices for optogenetic stimulation and optofluidic devices

All hardware and wireless devices for optogenetic stimulation with or without drug treatment were developed by and obtained from Neurolux (Urbana, IL)^68,69^. The implantation procedure was performed 1 week before optogenetic stimulation and consisted of injecting the needle portion of the device into brain and for subdermally implanting the body of the device on top of the skull. A custom mounting fixture for the device was used to connect to the arm of a stereotaxic stage for holding the needle and the body of the device. A small amount of medical device adhesive (Prism Medical Device Adhesive, 4541, Loctite) was applied near the point of insertion to fix the needle to the skull. Removing the holder on the fixture released the device body that was bent horizontally and glued to the skull before placing the skin over and closing it with Kwik-sil tissue glue. The optofluidic device was affixed to the skull using dental cement (Stoelting).

For optogenetic fear reinstatement, instead of exposure to a reminder shock, mice were exposed to a wirelessly powered blue light photostimulation (473 nm, 3 min, tonic, 7 W) using 2 ms 4 Hz or 20 Hz pulses. In one experiment, 4 Hz stimulation was performed using optofluidic wireless devices allowing for delivery of an N-methyl-D-aspartate receptor antagonist 2-amino-5-phosphonopentanoic acid (APV) or VEH (artificial cerebrospinal fluid).

For optogenetic reinstatement of CPP, instead of treatment with a priming dose of morphine, mice were exposed to 2 ms 4 Hz, 8 Hz, or 20 Hz pulses. In one experiment optogenetic stimulation at 20 Hz was co-applied during M-CPP over 15 min. For combined drug delivery and optogenetic stimulation, 1 h before stimulation, the chambers of the optofluidic device were filled with APV or VEH and 250 µl of the solution were delivered 40 min later.

For analyses of neuronal activity using cFos immunohistochemistry, we used naïve mice or mice exposed to a reminder shock, optogenetic stimulation with Hz, or optogenetic stimulation in the presence of APV. A pilot experiment demonstrated no significant effects in the AAV-GFP + 4 Hz group when compared with the naïve group (*t*_14_ = 0.8367, *P* = 0.4168), therefore this group was not used in follow up experiments. Mice were sacrificed 1 h later and tissue was processed as described below.

### Immunohistochemistry and immunofluorescence

Mice were anesthetized with an i.p. injection of 240 mg/kg Avertin and transcardially perfused with ice-cold 4% paraformaldehyde in phosphate buffer (pH 7.4, 150 mL per mouse). Brains were removed and post-fixed for 48 h in the same fixative and then immersed for 24 h each in 20 and 30% sucrose in phosphate buffer. Brains was frozen and 50 µm sections were cut for use in free-floating immunohistochemistry^70^ with primary antibodies against mCherry (1:1000, rabbit, Abcam AB167453 or 1:16000, chicken, ab205402), TH (1:2000, Immunostar 22941) or cFos (1:2000, Protein Tech, 26192-1-AP). Secondary antibodies were obtained from Jackson ImmunoResearch [1:300 each, Alexa Fluor^®^ 488 AffiniPure Donkey Anti-Rabbit IgG (H+L) and Alexa Fluor^®^ 594 AffiniPure Donkey Anti-Mouse IgG (H+L)] or Vector (1: 300, Biotinylated-anti chicken IgG). Sections were mounted using Vectashield (Vector) and observed with a confocal laser-scanning microscope (Olympus Fluoview FV10i). For light microscopy, signals were visualized with diaminobenzidine (Sigma).

### Image analysis and quantification

For cFos analyses, flat images were obtained using a 10× objective on a Leica microscope with a Leica DFC450 C digital camera. Leica Application Suite software was used for image capturing. Equal light exposure was applied to all captures. Image J was used for image analyses. All images were converted to binary format, and watershed segmentation was used to separate overlapping nuclei. For each section, background was calculated as a difference between signals from unlabeled tissue relative to corpus callosum and used as the threshold. Cell counts from hippocampal subregions were performed using two sections per mouse. Cells were defined by signal diameter >5 μm and intensity above 0.47 (arbitrary units). Counts were then normalized to area. Quantification of VTA-infected neurons was performed using the same approach and included the rostral VTA. Quantification of DH terminals in the hilus of the DG was performed with densitometry using the same area size for each section. For each section, background was calculated as a difference between signals from unlabeled tissue relative to corpus callosum and subtracted from the optical density. All quantifications were completed by an experimenter blind to treatment condition.

### Electrophysiological slice recordings

Four weeks after viral injection into the VTA, male and female mice were anesthetized with isoflurane gas in designed chamber. The brain was removed from the skull in ice-cold ACSF solution (containing (in mM): 125 NaCl, 25 NaHCO_3_, 2.5 KCl, 1.25 NaH_2_PO_4,_ 25 glucose, 2 CaCl_2_, 1 MgCl_2_, saturated with 95% O_2_ and 5% CO_2_) with 3 mM kynurenic acid and cut just caudal to bregma. A total of 300 µm thick coronal, slices were cut using a vibro-slicer (Leica VT-1200), stored for ~20 min at 35 °C and allowed to recover at room temperature (22–24 °C) for at least 30 min in the same solution, but without kynurenic acid.

For recordings, slices were transferred to a recording chamber and continuously superfused with ACSF (without kynurenic acid). Bath temperature was kept between 31 and 32 °C using a TC-324B control unit (Warner Instruments). DG granule cells of the DH were visualized using an upright microscope with oblique illumination and video microscopy using a digital camera (DVC). Cells were identified according to location, size, and shape. All recordings were performed using an Axopatch 200B amplifier. Signals were filtered at 2 kHz and sampled at 5 kHz, and data were acquired using pClamp9 software running on a PC. Pipettes were pulled from thick wall borosilicate glass with filament (1.5 mm OD; Sutter Instrument) using a horizontal puller (P97; Sutter Instruments) and were filled with a KCl internal solution consisting of (in mM): 148 KCl, 6 NaCl, 2 MgATP, 0.2 Na_3_GTP, 10 HEPES, 0.1 EGTA, 10 QX-314, pH 7.3 (with KOH). Pipette resistances in the working solutions ranged from 4 to 6 MΩ, yielding series resistances of 20–30 MΩ.

Cells were voltage-clamped at −70 mV. ChR2 stimulation was achieved delivering 1 ms light pulses (using a 450 nm LED light source; Prizmatix) directed to the slice through a 60×, 0.9NA water immersion objective. The light intensity was normalized in the different recordings. For each recorded cell it was first established the lowest intensity that would produce a maximal (saturating) response using a 1 ms pulse. This light intensity was then used throughout the experiment. In each cell we first recorded the synaptic response in absence of any synaptic blocker, and then in the presence of either picrotoxin (to measure glutamate currents) or kynurenic acid (to measure GABA currents). DAMGO was then added to the bath. In a subset of recordings picrotoxin (for GABA currents) or kynurenic acid (for glutamatergic currents) was finally added to confirm the identity of the recorded current. Current amplitudes were measured as the average (4–7 sweeps) peak response to a single light pulse. A 20–30 s-long interval between each stimulation was used to allow for presynaptic recovery and to avoid inducing long lasting synaptic plasticity. Current amplitudes were measured as the peak to baseline immediately preceding the stimulus from the average of 5–7 consecutive sweeps. Traces presented in the figures also represent the average of up 5–7 sweeps. Decay time constant was measured fitting a single exponential function over a 70 ms time window starting at the peak of the current.

To test the effectiveness of hM4D(Gi) in silencing synaptic transmission in slices, AAV8-DIO-hM4D(Gi), and AAV5-DIO- hChR2 were infused into DH of either vGlut1- or vGlut2-Cre mice via cannula. The infusion of hM4D(Gi) preceded that of hChR2 by 3 weeks, and 3 more weeks were then allowed for viral expression; thus, hM4D(Gi) was expressed for 6 weeks to be consistent with expression time used in behavioral experiments. DH slices were then prepared, and whole-cell recordings were made from DG granule cells. Photostimuli were delivered every 30 s to depolarize vGlut1^+^ or vGlut2^+^ DH terminals, evoking excitatory synaptic transmission detected as EPSCs in the recorded postsynaptic neuron. After 5 min of stable baseline recording, CNO (0.03 μM or 0.1 μM) was bath-applied and recording was continued for at least 5 min. Data were normalized to the mean baseline to assess the time-dependent effect of CNO at each concentration. To compare pre- and post-CNO EPSCs, traces from 1 min immediately before and the fifth minute after CNO (0.1 μM) application were averaged, and 50 ms mean current over a poststimulus interval of 50 ms was calculated.

All drugs were bath applied. Picrotoxin (Tocris, 100 mM stock) was dissolved in dimethylsulfoxide and stored at −20 °C. Kynurenic acid stock (Sigma; 500 mM) was dissolved into 1 N NaOH and kept at 4 °C. Fresh working solutions were prepared on the day of the experiment. DAMGO [(D-Ala2, N-Me-Phe4, Gly5-ol)-Enkephalin, Tocris] stock solution was 1 mM (in H_2_O) and was used at a final concentration 10 µM. Final concentrations used were: 50 μM for picrotoxin, 3 mM for kynurenic acid. A 1 mM stock of CNO solution was made before its bath application from powder (Sigma-Aldrich, C0832-5MG) using H_2_O as a solvent.

### Quantification and statistical analyses

Statistical analyses were performed using Graphpad. All statistical tests were two-sided. All samples were normally distributed, as determined with a one-sample Kolmogorov–Smirnov test. Homogeneity of variance was confirmed with Levene’s test for equality of variances. Data used for analyses included: (1) mice with virus expression in the VTA of 50% or more of the mean number of VTA-infected neurons across all experiments, (2) mice with detectable VTA-originating terminals in DH (in four cases there were no detectable signals despite string VTA labeling and these data were excluded), (3) mice with correctly implanted cannulas in DH), and (iv) mice showing over 80% preference for a CPP chamber before training (five cases). For the behavioral studies, two-way ANOVA using treatment and test as factors was used to determine: (1) changes of freezing relative to the last extinction test (for fear reinstatement), (2) changes of preference index and activity relative to CPP (for M-CPP), and (3) between group effects of treatments in all experiments. Significant *F* values were followed by post hoc multiple comparisons using Tukey’s test. Differences in the fear reinstatement index were also determined for treatment (CNO or VEH) as a factor using two-tailed Student’s *t* tests or one-way ANOVA. Statistical differences were considered significant for all *P* values < 0.05. Sex differences were determined using three-way ANOVA with factors sex, treatment, and test (for fear reinstatement and M-CPP) or Sex and treatment (for reinstatement and preference indices). For two- or three-way ANOVA, multiplicity adjusted *P* values accounting for multiple comparisons are reported. Due to limited availability of wireless optogenetic devices, indicated stimulation experiments were performed in the same group of mice on consecutive days and analyzed with repeated measures ANOVA. Group sizes were determined based on power analyses assuming a moderate effect size of 0.5 in previous experiments^71^. All significant behavioral effects were confirmed in at least two replicates, and the main effects of vGlut2-Cre silencing on fear reinstatement and M-CPP were replicated three times for each sex. Immunohistochemical findings were replicated multiple times because all brains were collected and immunostained with mCherry after each experiment. The sample sizes for the electrophysiological recordings was between 5 and 8 cells/group. Each data set (group) was obtained from three different animals, with the only exception of the data for DAMGO effect on glutamate current in males which were obtained from two different animals. In order to avoid potential recording bias, recordings from one male and one female animal were performed on the same day with alternating sequence on consecutive days. The data were analyzed with two-tailed *t*-tests or ANOVA. Nested ANOVA was used to determine the potential contribution of individual mice to the main effects. All data are presented as mean ± SEM with overlaid values for individual mice. Details of statistical analyses are found in figure legends.

### Reporting summary

Further information on research design is available in the Nature Research Reporting Summary linked to this article.

## Supplementary information

Supplementary Information

Reporting Summary

## Data Availability

All source data for the preparation of graphs (Figs. 2–7 and Supplementary Figs. 6, 7, 9–11) are provided in a linked Source Data Excel file.

## Source Data

Source Data
