# Supplementary material for: Excitatory VTA to DH projections provide a valence signal to memory circuits
Source: Nat Commun. 2020 Mar 19;11:1466. doi: 10.1038/s41467-020-15035-z (PMC7081331; doi:10.1038/s41467-020-15035-z)
Supplement: Supplementary file 1 — Supplementary Information [file 41467_2020_15035_MOESM1_ESM.pdf]

Excitatory VTA to DH projections provide a valence signal to memory circuits

Yuan et al.

Supplementary figures and figure legends

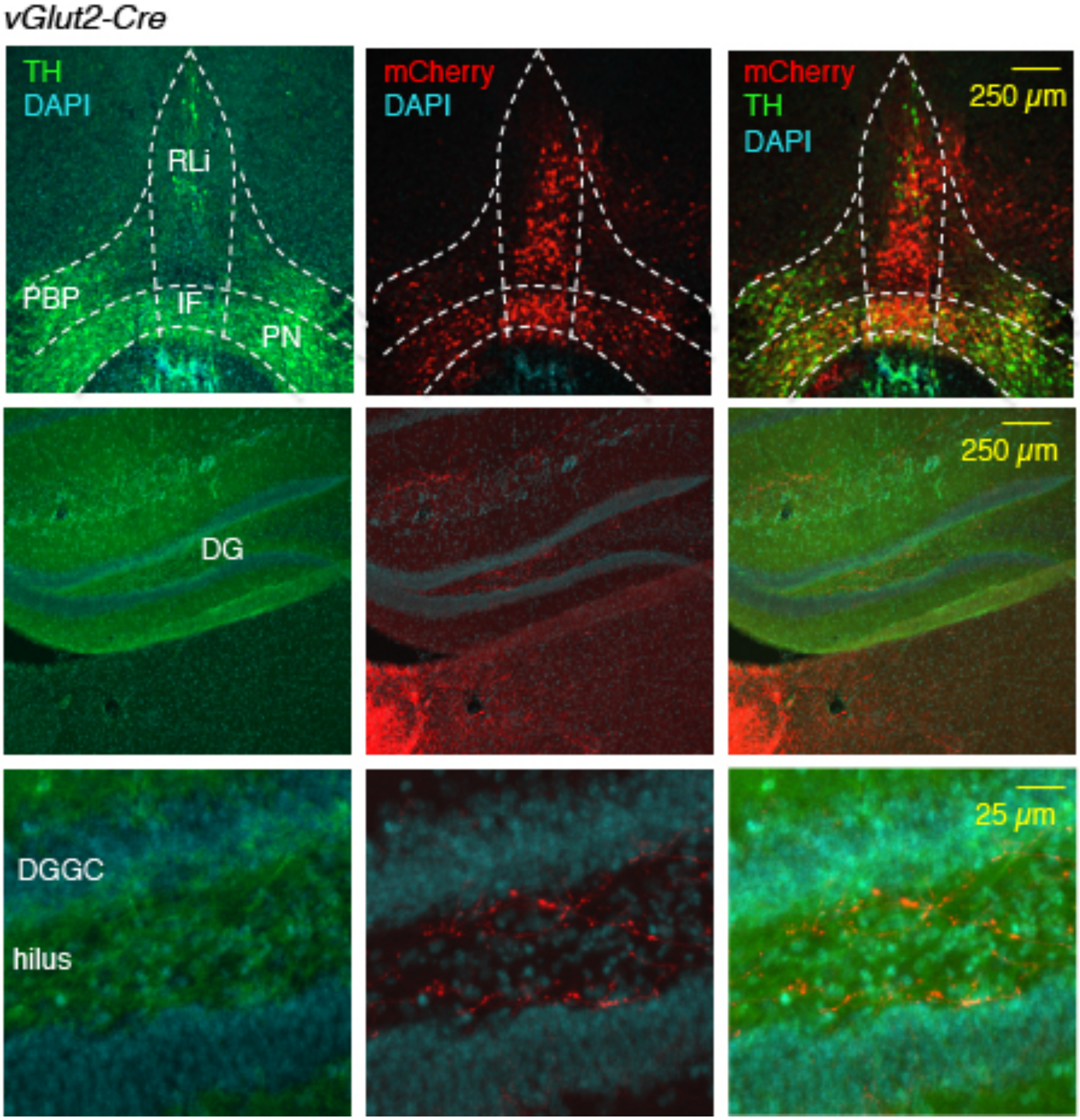

Supplementary Figure 1. Co-labeling with mCherry and TH in VTA and DH of vGlut2-Cre mice injected with AAV8-DIO-mCherry. Relationship between glutamatergic and TH-positive neurons in the VTA (top) and terminals in DH (middle). High magnification images of the dentate gyrus are shown at the bottom.

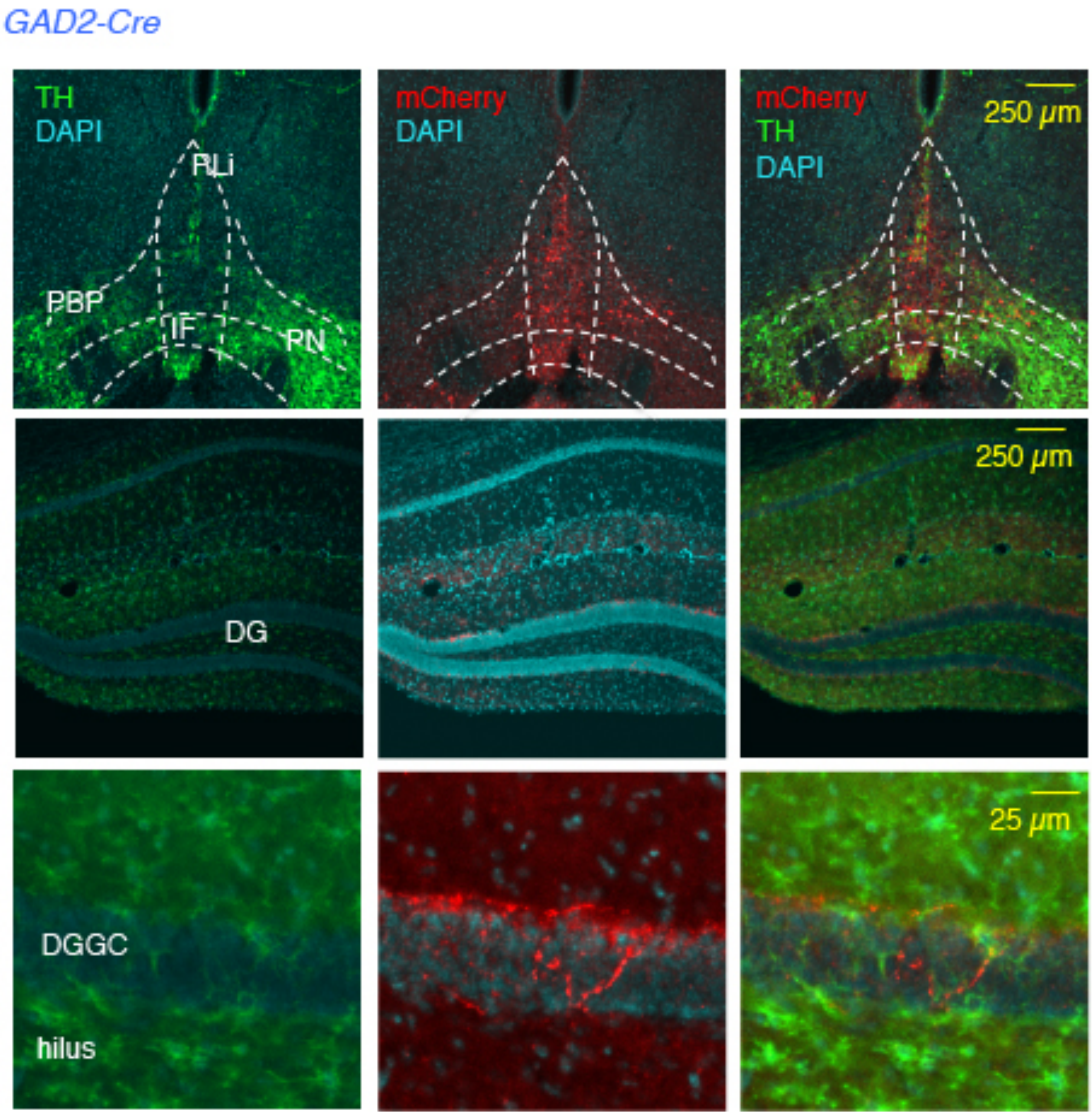

Supplementary Figure 2. Co-labeling with mCherry and TH in VTA and DH of GAD2-Cre mice injected with AAV8-DIO-mCherry. Relationship between GABAergic and TH-positive neurons in the VTA (top) and terminals in DH (middle). High magnification images of the dentate gyrus are shown at the bottom.

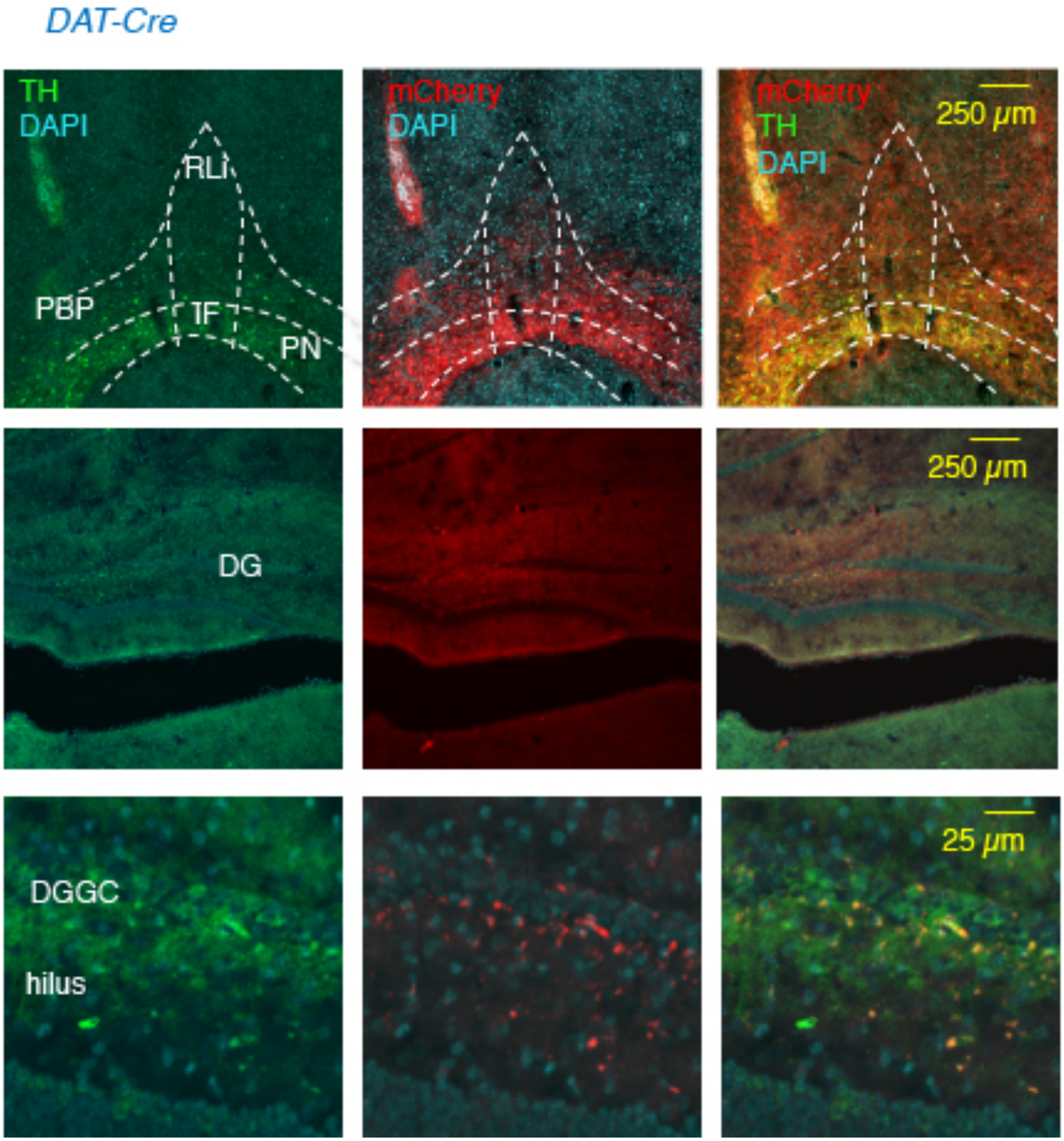

Supplementary Figure 3. Co-labeling with mCherry and TH in VTA and DH of DAT-Cre mice injected with AAV8-DIO-mCherry. mCherry and TH-positive neurons in the VTA (top) and terminals in DH (middle) in DAT-Cre male mice injected with AAV8-DIO-mCherry. High magnification images of the dentate gyrus are shown at the bottom.

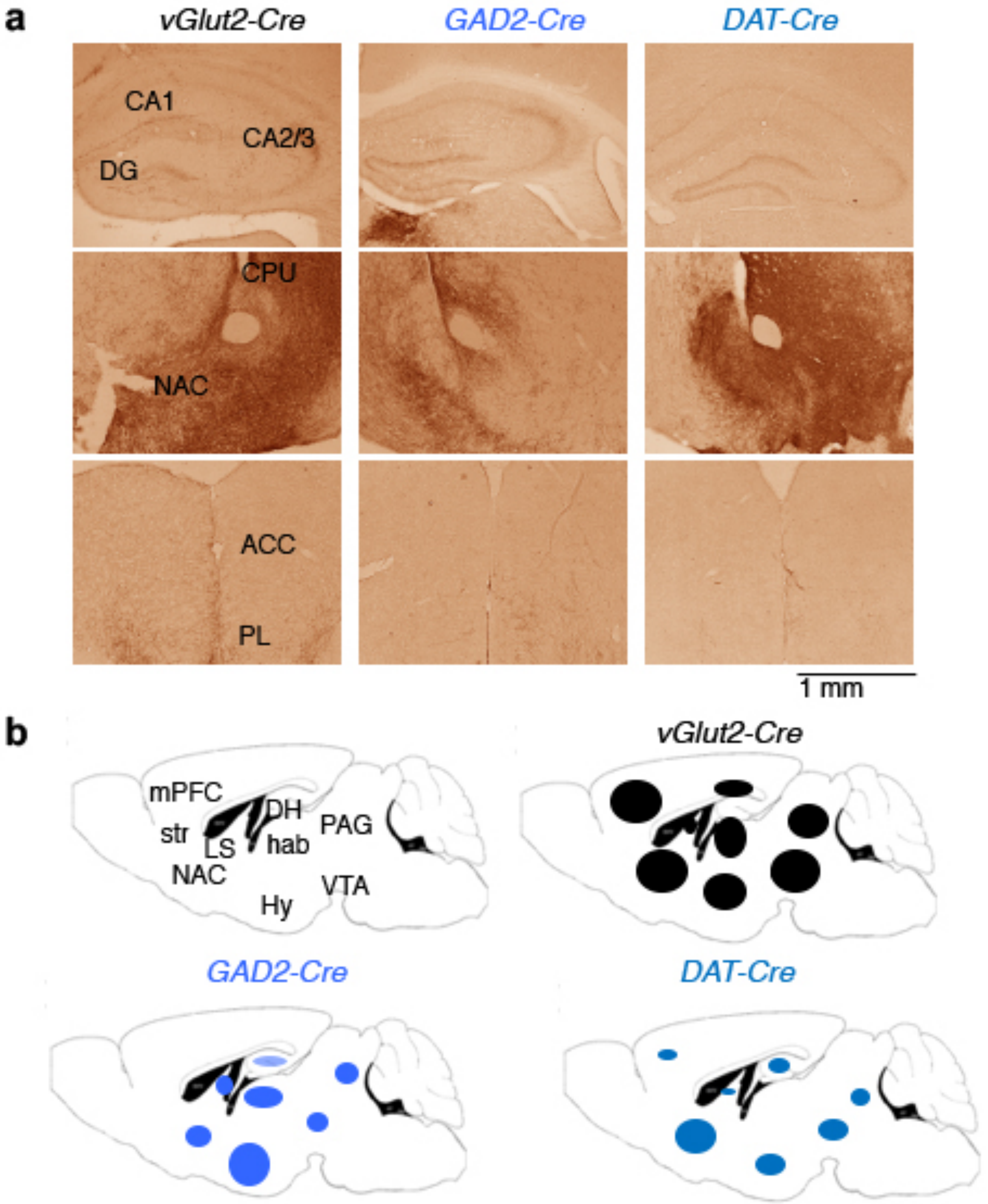

Supplementary Figure 4. Summary of main glutamatergic, GABAergic, and dopaminergic VTA DH projections. **a** VTA terminals visualized with mCherry immunohistochemistry in the dorsal hippocampus, nucleus accumbens, and prefrontal cortex. **b** Schematics summarizing histological analyses in vGlut2-Cre, GAD2-Cre, and DAT-Cre mice.

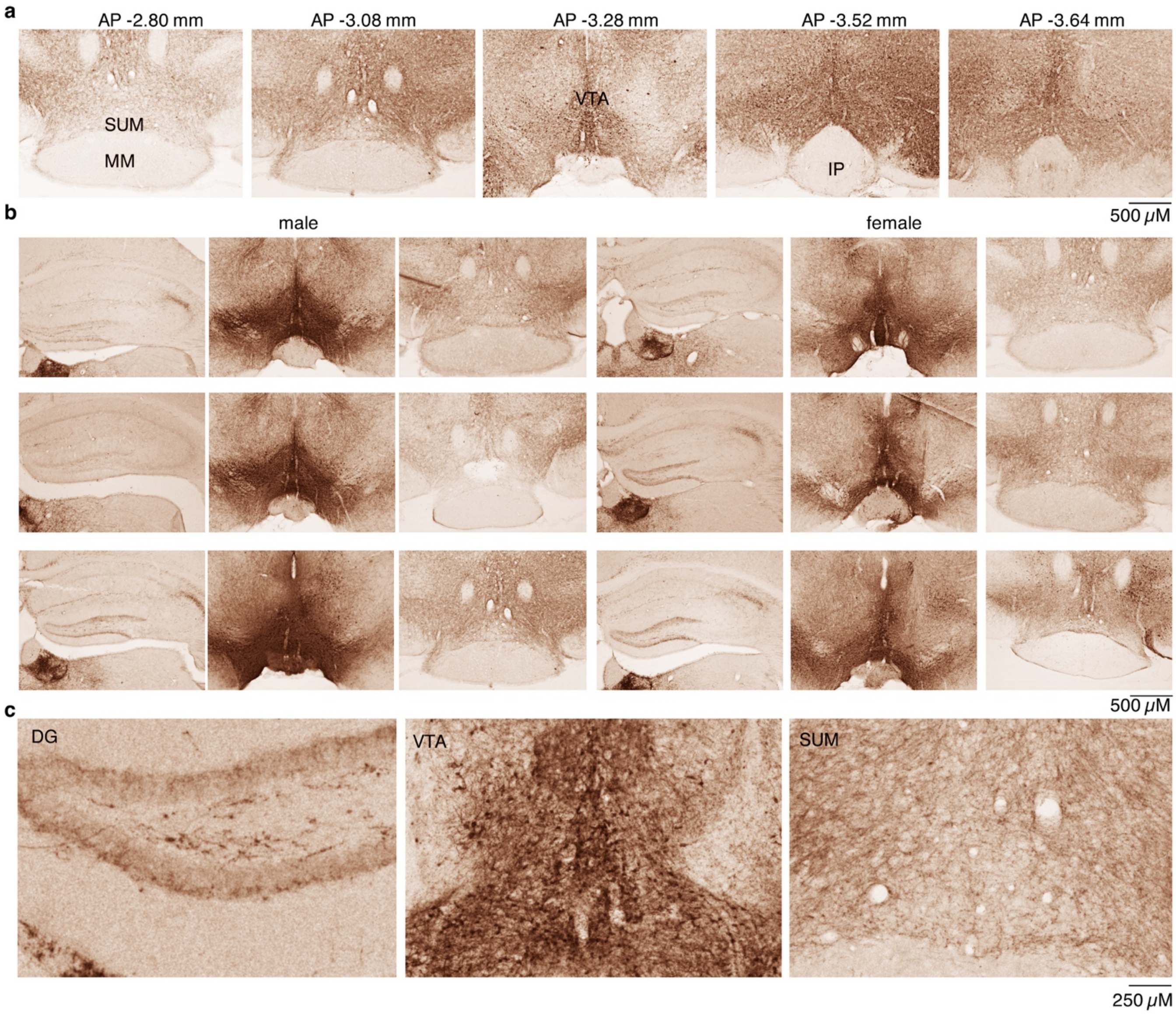

Supplementary Figure 5. Spread of the AAV-DIO-hM4(Gi) DREADD. **a** Images demonstrating the spread of viruses after infusion in VTA from coordinates AP -2.8mm to -3.64 mm posterior to the bregma. **b** Images demonstrating mCherry signals in DH, VTA, and SUM of male and female mice. Only fibers of passage are found in SUM. **c** High magnification images illustrating mCherry staining in DH, VTA, and SUM.

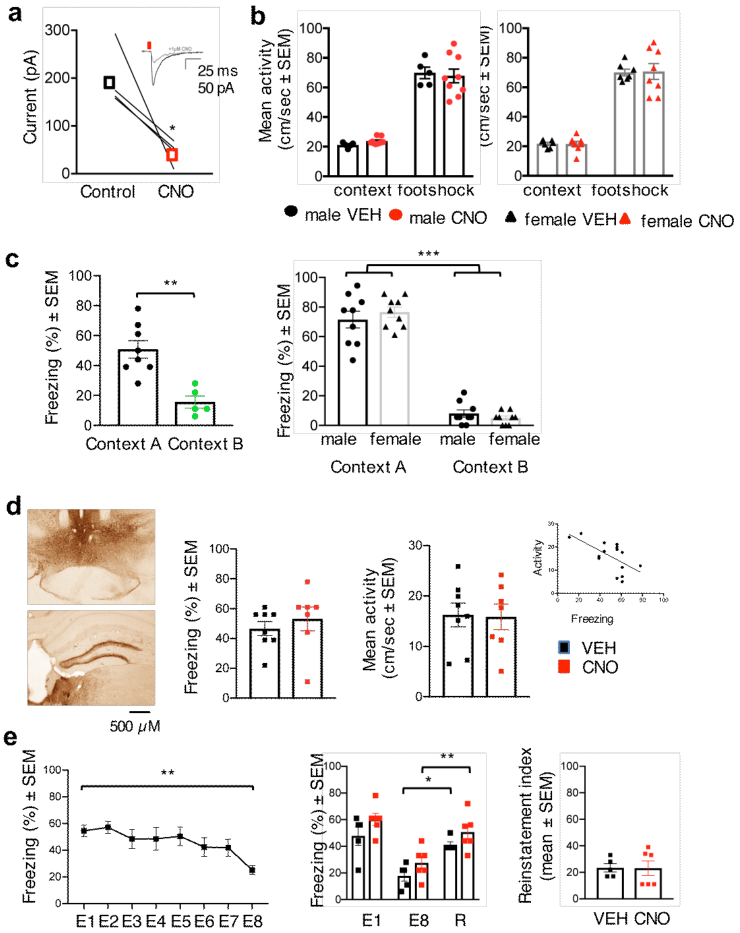

Supplementary Figure 6. Specificity controls for pharmacological and behavioral experiments. a CNO reduced current amplitudes in response to optogenetic stimulation of VTA→DH excitatory afferents (paired two-tailed t-test, $t_3 = 3.468$, **P* = 0.0404). **b** CNO did not affect activity to context (two -tailed unpaired *t* test, males: $t_{12} = 1.714$, *P* = 0.1123; females: $t_{13} = 0.06587$, *P* = 0.9485) or footshock (males: $t_{12} = 0.2903$, *P* = 0.7766; females: $t_{13} = 0.2389$, *P* = 0.8149) during training (VEH males *n* = 5, CNO males *n* = 9, VEH females *n* = 7, and CNO females *n* = 8). **c** Left, context specificity of CPFE, shown by freezing in mice preexposed to context A (*n* = 8) vs context B (*n* = 5), before immediate shock and testing in context A (unpaired two-tailed t-test, $t_{11} = 4.282$, ***P* < 0.0013). Right, context specific fear reinstatement in male (*n* = 9) and female (*n* = 9) mice [two-way ANOVA with Context and Sex as factors, Context effect, $F_{(1, 32)} = 346.2$, ****P* < 0.0001]. **d** Immunostaining of DGGC (left bottom) after virus infusion in SUM (left top). Silencing SUM→VTA terminals did not affect freezing (middle, $t_{13}= 0.7298$, *P* = 0.4784) and activity (right, $t_{13} = 0.1174$, *P* = 0.9083) in VEH (*n* = 7) and CNO (*n* = 8) groups, as determined by unpaired two-tailed t-test. Inset: correlation between activity and freezing (*r* = -0.6406, *P* = 0.0101). **e** Left, extinction curve [RM one-way ANOVA, $F_{(3.013, 30.13)} = 5.553$, ***P* = 0.0037]. Middle, freezing during the first context test (E1), last extinction test (E8), and reinstatement test in mice injected with VEH (*n* = 5) or CNO (*n* = 6) before the reminder shock. There was no Treatment x Test interaction [two-way ANOVA $F_{(2, 27)} = 0.0$, *P* = 0.9481]. Fear reinstatement was found both in the VEH (**P* = 0.0357) and CNO (***P* = 0.0189) groups]. Right, CNO did not affect the fear reinstatement index (right, two -tailed unpaired *t* test, $t_9 = 0.03476$, *P* = 0.973).

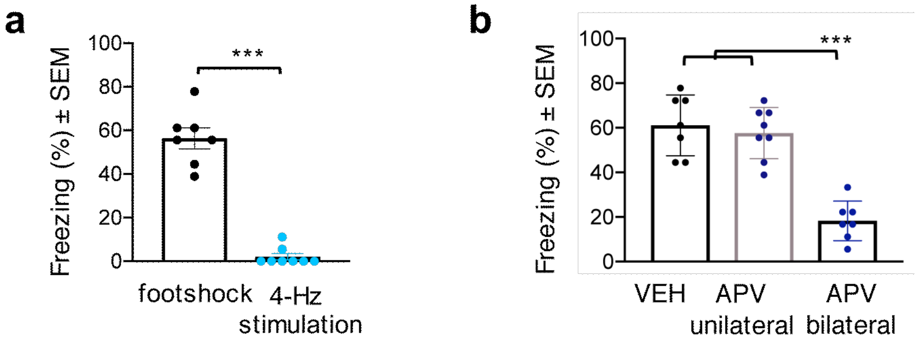

Supplementary Figure 7. Specificity controls for experiments with optogenetic stimulation. a Optogenetic stimulation alone (*n* = 7) could not substitute for exposure to footshock (*n* = 8) during the first context exposure, as revealed by lack of freezing behavior in mice during the subsequent context test (two-tailed unpaired t-test, t_{13} =11.54, ****P* < 0.0001), although the same stimulation (3 min, tonic, 4 Hz) was effective in reinstating freezing behavior. **b** Unilateral infusion of APV (*n* = 8) did not affect freezing relative to VEH (*n* = 7, three receiving vehicle bilaterally and four unilaterally) whereas bilateral infusion of APV (*n* = 7) significantly impaired freezing [one-way ANOVA $F_{(2, 19)}$ = 30.54, ****P* < 0.0001].

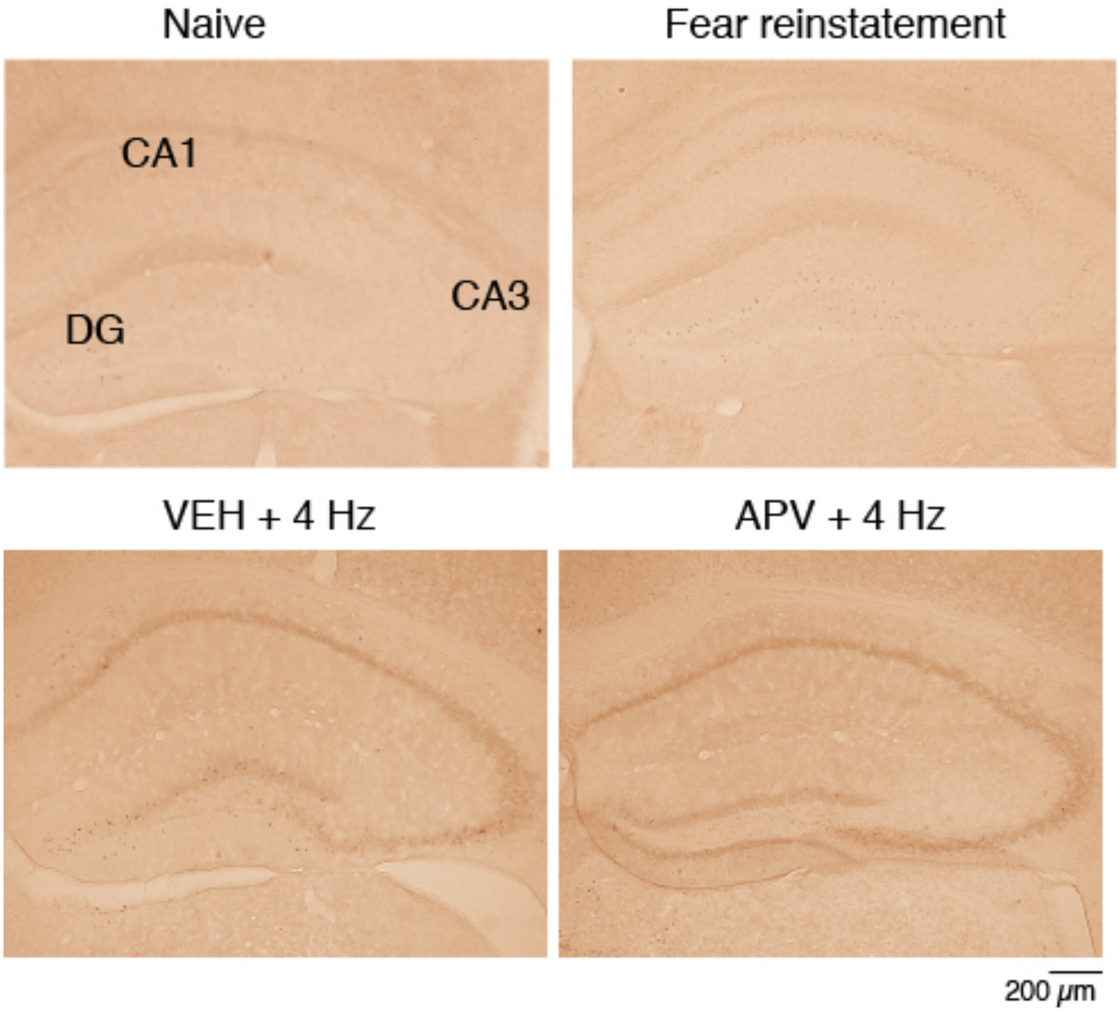

Supplementary Figure 8. cFos levels induced in DH by optogenetic stimulation of glutamatergic VTA terminals. Low magnification images of cFos activation in DH after reminder shock or optogenetic stimulation.

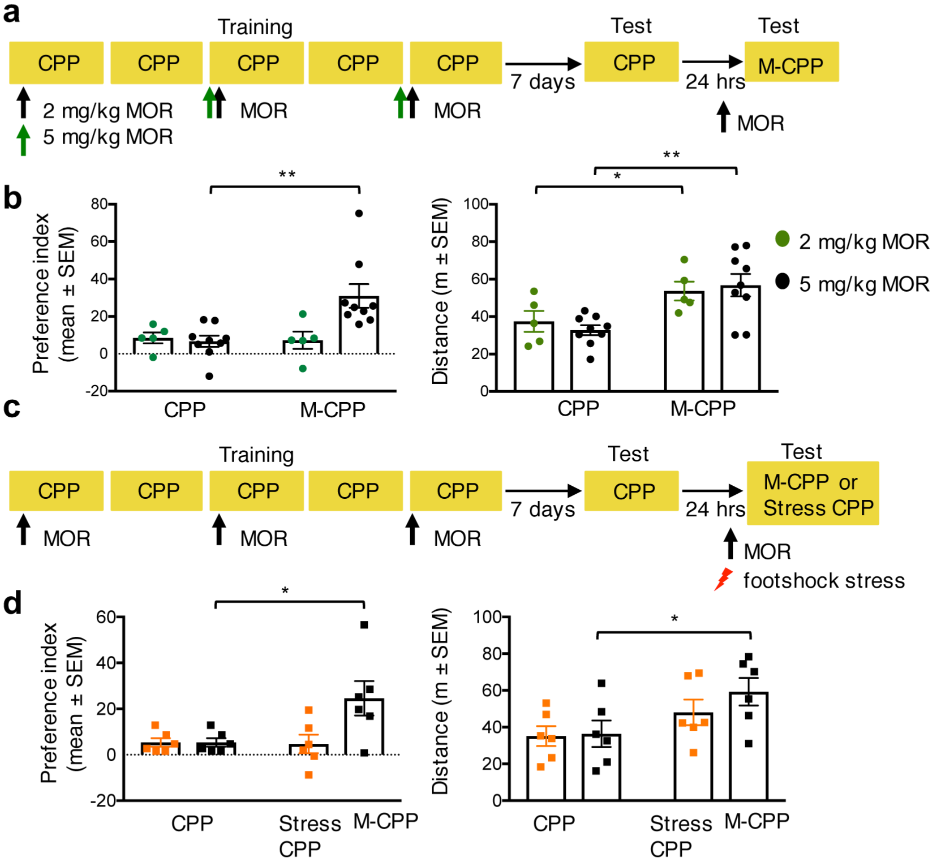

Supplementary Figure 9. Effects of low dose of morphine and unpredictable stress on CPP. a Paradigm for assessing the reinforcing effects of 2 mg/kg morphine treatment. **b** The priming dose of morphine (2 mg/kg) was not effective in supporting CPP, as revealed by significant dose x test interaction [left, two-way ANOVA, $F_{(1, 24)} = 6.119$, $P < 0.0208$, Tukey's multiple comparisons test $P = 0.9985$ 2 mg/kg morphine, $^{**}P = 0.0033$ 5 mg/kg morphine]. Although significant effect was found on locomotor activity [right, $F_{(1,24)} = 14.83$, $P = 0.0008$], this effect was less pronounced when mice were trained with 2 mg/kg ($^{*}P < 0.0430$) rather than 5 mg/kg ($^{**}P < 0.0040$) of morphine. The number of mice/group was $n = 5$ (2 males, 3 females) for 2 mg/kg morphine and $n = 9$ (4 males, 5 females) for 5 mg/kg morphine. **c** Paradigm for testing stress effects on CPP. **d** Exposure to unpredictable footshocks (15 2-s shocks over 15 min, $n = 6$, 3 males, 3 females) did not induce CPP following 7-day morphine abstinence [two-way ANOVA , $F_{(1, 20)} = 4.997$, $P = 0.0370$, Tukey's multiple comparisons test, stress $P = 0.5523$] whereas morphine ($n = 6$, 3 males, 3 females) was effective both in enhancing preference scores ($^{*}P = 0.0289$) and in inducing enhanced locomotion [two-way ANOVA, $F_{(1,10)} = 11.81$, $P = 0.0084$, stress $P = 0.4802$, morphine $^{*}P = 0.0239$].

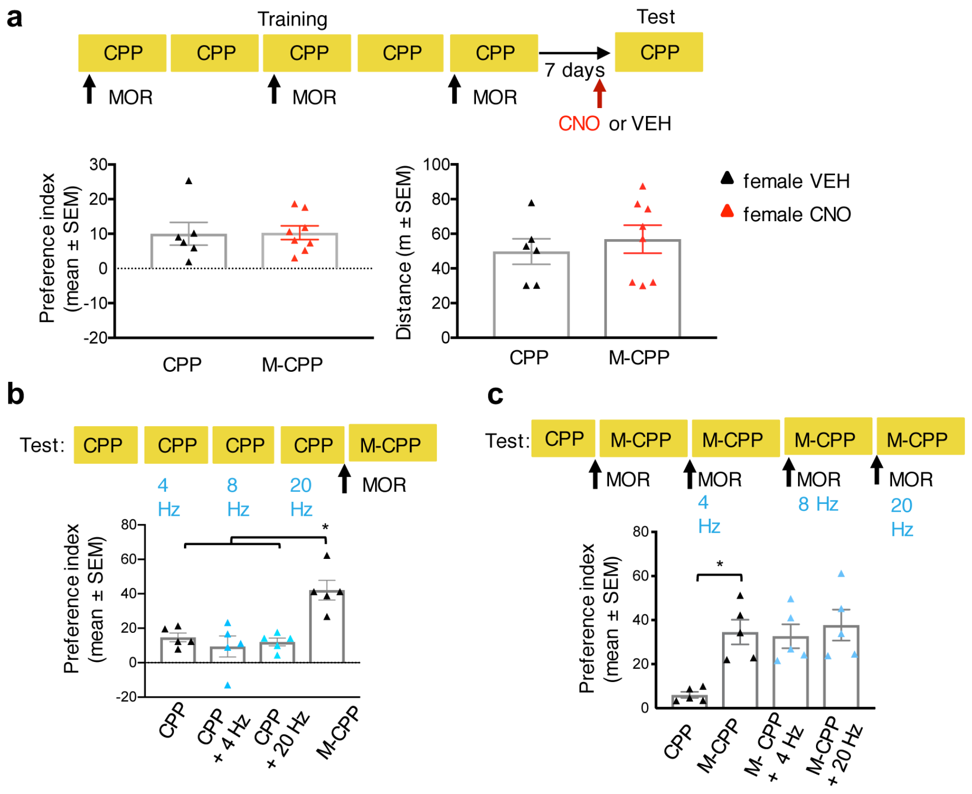

Supplementary Figure 10. Silencing or stimulation of glutamatergic VTA-DH terminals does not affect CPP or M-CPP in vGlut2-Cre female mice. a CNO did not affect CPP [two -tailed unpaired *t* test, $t_{12} = 0.0856$, $P = 0.9332$, CNO ($n = 8$) vs. vehicle ($n = 6$)] or activity in the CPP box (two -tailed unpaired *t* test, $t_{12} = 0.6257$, $P = 0.5432$) in the absence of morphine. **b** Optogenetic stimulation of 5 females on consecutive days with 4, 8, 20 Hz, or morphine during CPP revealed a significant effect of Treatment [repeated measures ANOVA $F_{(1.975,\ 7.902)} = 13.72$, $P = 0.0027$, however posthoc tests revealed significant effects only for M-CPP relative to CPP (*$P = 0.0340$), whereas stimulation was ineffective (4 Hz $P = 0.8879$), and 20 Hz ($P = 0.8871$)]. **c** Optogenetic stimulation of 5 females on consecutive days with 4, 8, or 20 Hz during M-CPP was also ineffective relative to M-CPP alone [repeated measures ANOVA $F_{(1.207,\ 4.828)} = 16.94$, $P = 0.0088$, M-CPP vs CPP *$P = 0.0323$, vs 4 Hz $P = 0.8569$, and vs 20 Hz $P = 0.03948$].

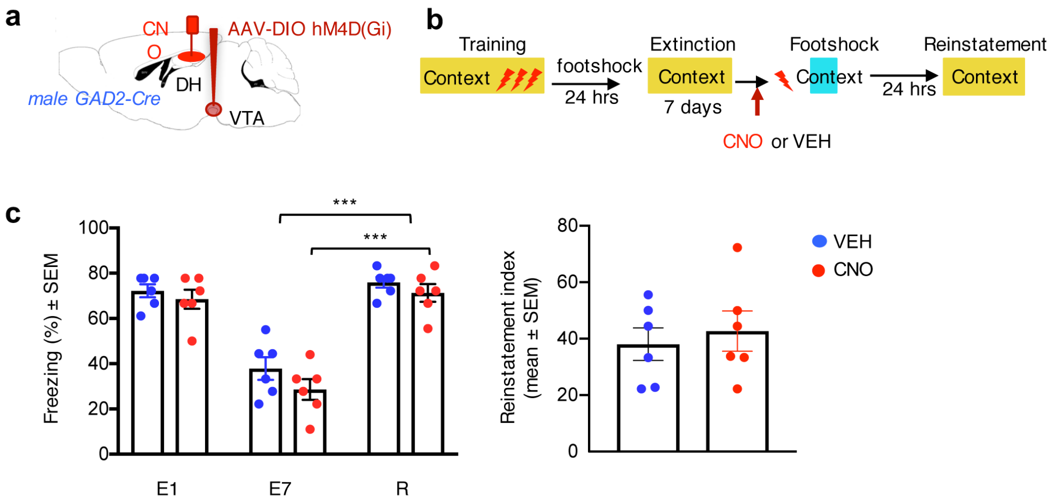

Supplementary Figure 11. CNO does not affect fear reinstatement of GAD2-Cre mice trained with exposures to three footshocks. a Sagittal schematic of virus infusion into the VTA and cannulation in the DH. **b** Diagram of the behavioral paradigm. Mice trained with three footshock presentations (2 s, 0.7 mA, constant current) in the context (3 min) were subsequently exposed to the context only (3 min, every 24 hr) until freezing behavior was reduced over successive tests. Footshock reminder was presented immediately after placement in a novel context and mice were re-tested for reinstatement of fear in the conditioning context the following day. **c** Mice were injected with VEH (n = 6) or CNO (n = 6) one hour before the reminder shock. Comparison of freezing behavior in male mice during the first context test (E1), last extinction test (E7), and reinstatement test revealed no effects of CNO on fear reinstatement [left, two-way ANOVA, Treatment $F_{(1, 30)} = 3.335$, $P = 0.0778$, Tukey's multiple comparisons test VEH ***$P < 0.0001$, CNO ***$P < 0.0001$] or on the fear reinstatement index (right, two -tailed unpaired t test, $t_{10} = 0.5056$, $P = 0.6241$).

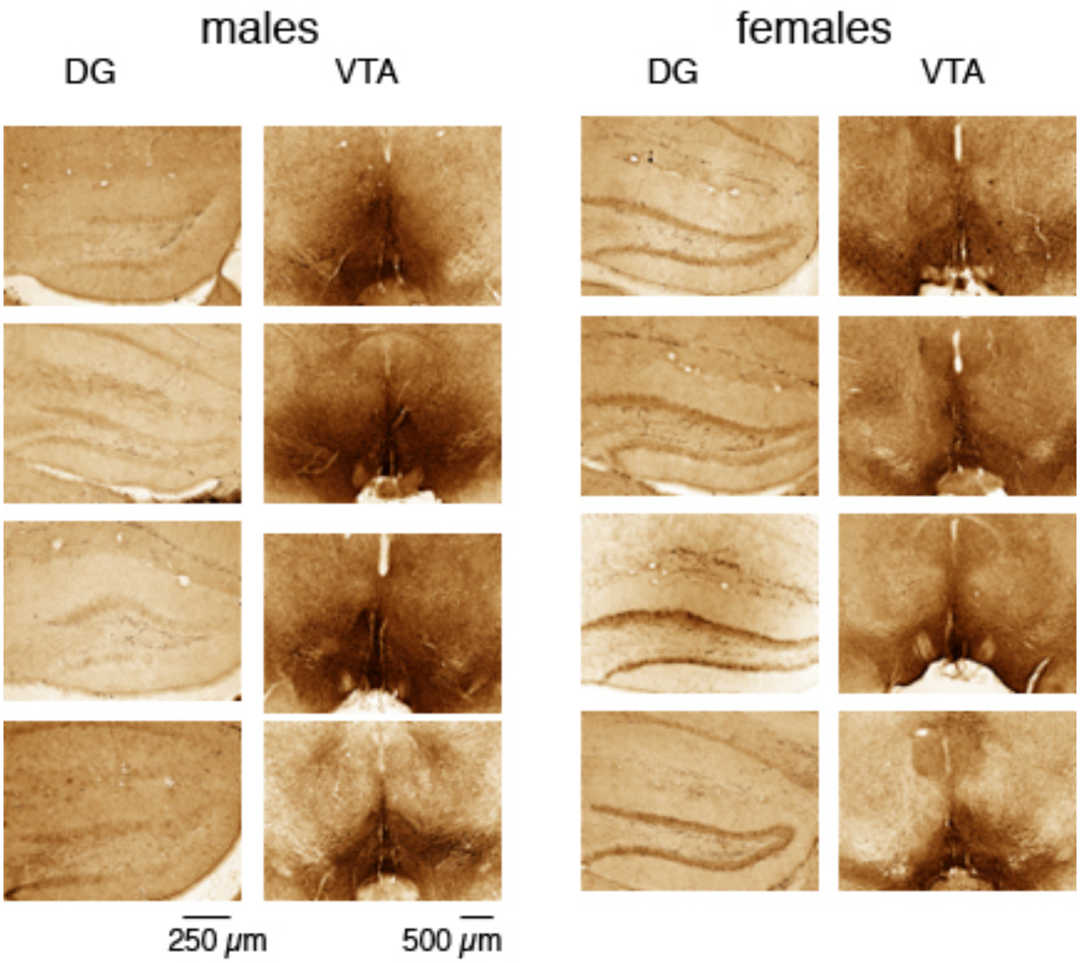

Supplementary Figure 12. vGlut2-positive DH terminals stemming from VTA in males and females. Typical mCherry signals in DH and VTA of vGlut2-Cre male and female mice injected with AAV8-DIO-mCherry in the VTA.
