# Supplementary material for: Excitatory VTA to DH projections provide a valence signal to memory circuits
Source: Nat Commun. 2020 Mar 19;11:1466. doi: 10.1038/s41467-020-15035-z (PMC7081331; doi:10.1038/s41467-020-15035-z)
Supplement: Supplementary file 2 — Reporting Summary [file 41467_2020_15035_MOESM2_ESM.pdf]

nature research

Corresponding author(s): Jelena Radulovic

Last updated by author(s): Jan 30, 2020

Reporting Summary

Nature Research wishes to improve the reproducibility of the work that we publish. This form provides structure for consistency and transparency in reporting. For further information on Nature Research policies, see Authors & Referees and the Editorial Policy Checklist .

Statistics

For all statistical analyses, confirm that the following items are present in the figure legend, table legend, main text, or Methods section.

n/a	Confirmed	
☐	☒	The exact sample size (n) for each experimental group/condition, given as a discrete number and unit of measurement
☐	☒	A statement on whether measurements were taken from distinct samples or whether the same sample was measured repeatedly
☐	☒	The statistical test(s) used AND whether they are one- or two-sided<br>*Only common tests should be described solely by name; describe more complex techniques in the Methods section.*
☐	☒	A description of all covariates tested
☐	☒	A description of any assumptions or corrections, such as tests of normality and adjustment for multiple comparisons
☐	☒	A full description of the statistical parameters including central tendency (e.g. means) or other basic estimates (e.g. regression coefficient) AND variation (e.g. standard deviation) or associated estimates of uncertainty (e.g. confidence intervals)
☐	☒	For null hypothesis testing, the test statistic (e.g. F, t, r) with confidence intervals, effect sizes, degrees of freedom and P value noted<br>*Give P values as exact values whenever suitable.*
☒	☐	For Bayesian analysis, information on the choice of priors and Markov chain Monte Carlo settings
☒	☐	For hierarchical and complex designs, identification of the appropriate level for tests and full reporting of outcomes
☒	☐	Estimates of effect sizes (e.g. Cohen's d, Pearson's r), indicating how they were calculated

Our web collection on statistics for biologists contains articles on many of the points above.

Software and code

Policy information about availability of computer code

Data collection	TSE systems Inc.
Data analysis	Graphpad (Prism 8)

For manuscripts utilizing custom algorithms or software that are central to the research but not yet described in published literature, software must be made available to editors/reviewers. We strongly encourage code deposition in a community repository (e.g. GitHub). See the Nature Research guidelines for submitting code & software for further information.

Data

Policy information about availability of data

All manuscripts must include a data availability statement. This statement should provide the following information, where applicable:

- Accession codes, unique identifiers, or web links for publicly available datasets
- A list of figures that have associated raw data
- A description of any restrictions on data availability

A Source Data file is provided. A list of figures that have associated raw data is provided.

Field-specific reporting

Please select the one below that is the best fit for your research. If you are not sure, read the appropriate sections before making your selection.

☒ Life sciences ☐ Behavioural & social sciences ☐ Ecological, evolutionary & environmental sciences

For a reference copy of the document with all sections, see nature.com/documents/nr-reporting-summary-flat.pdf

Life sciences study design

All studies must disclose on these points even when the disclosure is negative.

Sample size	Group sizes were determined based on power analyses assuming a moderate effect size of 0.5 in previous experiments.
Data exclusions	Data used for analyses included: (i) mice with virus expression in the VTA of 50% or more of the mean number of VTA-infected neurons across all experiments, (ii) mice with detectable VTA-originating terminals in DH (in four cases there were no detectable signals despite string VTA labeling and these data were excluded), (iii) mice with correctly implanted cannulas in DH), and (iv) mice showing over 80% preference for a CPP chamber before training (five cases).
Replication	All behavioral experiments were replicated twice, and the main effects of vGlut2-Cre silencing on fear reinstatement and M-CPP were replicated three times for each sex. Immunohistochemical findings were replicated multiple times because all brains were collected and immunostained with mCherry after each experiment.
Randomization	Randomization was performed by assigning similar numbers of littermates to the different treatment conditions.
Blinding	The behavioral experiments were performed by two experimenters of which one was blind to genotypes and drug treatments.

Reporting for specific materials, systems and methods

We require information from authors about some types of materials, experimental systems and methods used in many studies. Here, indicate whether each material, system or method listed is relevant to your study. If you are not sure if a list item applies to your research, read the appropriate section before selecting a response.

Materials & experimental systems

n/a	Involved in the study	
☐	☒	Antibodies
☒	☐	Eukaryotic cell lines
☒	☐	Palaeontology
☐	☒	Animals and other organisms
☒	☐	Human research participants
☒	☐	Clinical data

Methods

n/a	Involved in the study	
☒	☐	ChIP-seq
☒	☐	Flow cytometry
☒	☐	MRI-based neuroimaging

Antibodies

Antibodies used	Primary antibodies against: mCherry (1:1000, rabbit, Abcam AB167453 or 1:16000, chicken, ab205402), tyrosine hydroxylase (1:2000, Immunostar 22941) or cFos (1:2000, Protein Tech, 26192-1-AP).<br>Secondary antibodies were obtained from Jackson ImmunoResearch [1:300 each, Alexa Fluor® 488 AffiniPure Donkey Anti-Rabbit IgG (H+L) and Alexa Fluor® 594 AffiniPure Donkey Anti-Mouse IgG (H+L)] or Vector (1: 300, Biotinylated-anti chicken IgG).
Validation	mCherry Abcam AB167453 : Validated in WB, IHC, ICC/IF. Cited in 71 publication(s). Independently reviewed in 12 review(s). Signals detected only in brain regions infused with mCherry carrying reproter virus and their projections.<br>mCherry ab205402: No signals detected in brain tissue not expressing mCherry carrying reproter virus.<br>TH, Immunostar 22941: Extensively used, labels predicted TH-positive neurons significantly above background.<br>cFos: rotein Tech, 26192-1-AP: KD/KO validated.<br>Secondary antibodies: Internal/in house validation using isotype controls. LAck of cross-reactivity with primary antibodies during immunofluorescent co-labelling.

Animals and other organisms

Policy information about studies involving animals; ARRIVE guidelines recommended for reporting animal research

Laboratory animals	We performed the experiments using male and female C57BL/6J mice, vGlut2-Cre, GAD2-Cre, and DAT-Cre mice. Wild type C57BL/6J mice were purchased from Harlan, Indianapolis, IN). All other mouse lines were obtained from the Jackson Laboratory (Bar Harbor, ME). The vGlut2-Cre knockin mice, also known as Slc17a6tm2(cre) and Lowl or VGlut2-ires-Cre, express Cre recombinase in excitatory glutamatergic neuron cell bodies containing vGlut2, as described previously 62. The GAD2-Cre mice (Gad2tm2(cre)Zjh/J), developed and characterized by Taniguchi et al. 63, express Cre in GABAergic neurons, whereas DAT-Cre mice [B6.SJL-Slc6a3tm1.1(cre)Bckmn/J], developed and characterized by Backman et al. 64, express Cre in dopaminergic neurons containing the dopamine transporter DAT. All mice were 8 weeks of age at the beginning of the experiments.
Wild animals	*Provide details on animals observed in or captured in the field; report species, sex and age where possible. Describe how animals were caught and transported and what happened to captive animals after the study (if killed, explain why and describe method; if released, say where and when) OR state that the study did not involve wild animals.*

Field-collected samples	*For laboratory work with field-collected samples, describe all relevant parameters such as housing, maintenance, temperature, photoperiod and end-of-experiment protocol OR state that the study did not involve samples collected from the field.*
Ethics oversight	All animal procedures used in this study were approved by the Northwestern University IACUC and complied with federal regulations set forth by the National Institutes of Health

Note that full information on the approval of the study protocol must also be provided in the manuscript.
